# *Saccharomyces boulardii* (CNCM I-745) improves intestinal damage in sepsis by remodeling intestinal flora structure

**DOI:** 10.3389/fcimb.2025.1602792

**Published:** 2025-07-31

**Authors:** Hui-Ying Liu, Yao Li, Yi-Lu Lin, Yu-Jia Tang, Jin-Da Zhao, Jia Xu, Kuo Wang, Ying-Fei Zhi, Yan Zhang, Jia-Le Deng, Kai Kang, Ying Chen, Yang Gao

**Affiliations:** ^1^ Department of Critical Care Medicine, The Sixth Affiliated Hospital of Harbin Medical University, Harbin, Heilongjiang, China; ^2^ Department of Critical Care Medicine, The First Affiliated Hospital of Jiamusi University, Jiamusi, Heilongjiang, China; ^3^ Department of Critical Care Medicine, The First Affiliated Hospital of Harbin Medical University, Harbin, Heilongjiang, China

**Keywords:** *Saccharomyces boulardii*, sepsis, gut microbiota, gut barrier dysfunction, 16S rRNA gene sequencing

## Abstract

**Background:**

Sepsis is one of the leading causes of mortality among intensive care unit (ICU) patients. The intestinal tract is the primary organ affected by sepsis, resulting in dysbiosis of the gut microbiota and negatively impacting long-term prognosis. This study investigated the protective effects of *Saccharomyces boulardii (S. boulardii)* on intestinal damage during sepsis.

**Methods:**

Distilled water was administered orally by intragastric for 4 weeks in control group and sepsis group. *S. boulardii* (CNCM I-745) suspension (10^8^CFU/ml) was administered orally by intragastric for 4 weeks in probiotic group and treatment group. Rats in control group and probiotic group received the intraperitoneal injection of normal saline (5ml/kg). Rats in sepsis group and treatment group received the intraperitoneal injection of LPS solution (1mg/ml). Eight hours after the intraperitoneal injection, samples of serum, colonic tissue, and colonic contents were collected and stored at - 80°C. Four weeks later, the samples of colonic contents were taken to observe the alterations in the intestinal microbiota.

**Results:**

Sepsis led to an increase in the expression of IL-6, TNF-α, and a decrease in the expression of occludin. After treatment with *S. boulardii* (CNCM I-745), the inflammatory damage with sepsis was reduced, and the expression level of occludin was significantly increased. High-throughput sequencing analysis revealed that sepsis injury led to a decline in both the diversity and abundance of the gut microbiota. Simultaneously, the colonization of beneficial bacteria within the intestine diminished, whereas the colonization of harmful bacteria surged. However, upon administration of *S. boulardii* (CNCM I-745), an increase in the diversity and abundance of the gut microbiota was evident. Moreover, the composition of the gut microbiota underwent a discernible alteration.

**Conclusion:**

Sepsis induces impairment of intestinal barrier function and exacerbates inflammatory responses. The use of *S. boulardii* (CNCM I-745) can modulate the composition of the intestinal flora by enhancing the colonization of beneficial bacteria while reducing the presence of harmful bacteria. It helps maintain intestinal mucosal barrier function, mitigates intestinal damage associated with sepsis, and potentially influences the long-term growth and development of pediatric sepsis patients.

## 1 Introduction

Sepsis stands as a prominent cause of mortality among pediatric patients admitted to the intensive care unit (PICU) (HHazwani et al., 2020). The dire prognosis associated with sepsis, coupled with its substantial financial burden, places considerable strain on both families and society at large. The pathogenesis and therapeutic management of sepsis have consistently garnered significant attention in research endeavors. Notably, the intestine emerges as the primary organ affected by sepsis. A robust intestinal barrier function and intact immune system are pivotal factors in maintaining a healthy intestinal microenvironment (Sun et al., 2023). A stable and healthy intestinal microecology is instrumental in mitigating intestinal injury. Various factors, including dietary habits (Frazier et al., 2022), abuse of antibiotics, infections, and environmental stressors (Zhang et al., 2022), can disrupt the delicate balance of intestinal flora (Albillos et al., 2020). In the context of sepsis, intestinal hypoperfusion, apoptosis of intestinal epithelial cells, a systemic cytokine storm, and dysregulation of intestinal flora collectively contribute to heightened intestinal epithelial permeability (Guo et al., 2024 and Gagnani et al., 2024). Bacterial infection stands as the primary etiological agent of sepsis, with antibiotic therapy serving as the cornerstone of its treatment. However, the administration of antibiotics not only elevates the risk of intestinal flora disturbance but also undermines the efficacy of probiotics. Emerging studies have demonstrated that probiotics exert an anti-inflammatory effect by modulating the immune system, bolstering intestinal barrier function, and producing metabolites such as short-chain fatty acids (SCFAs) (Oh et al., 2025 and Gurung et al., 2025 and Quinn-Bohmann et al., 2024). *S. boulardi*i (CNCM I-745), belonging to the fungal kingdom, within the *Saccharomyces* family and genus, exhibits minimal susceptibility to antibiotics. The organism thrives optimally at a temperature of 37°C, mirroring the normal human body temperature. By occupying intestinal mucosal binding sites, *S. boulardi*i (CNCM I-745) effectively thwarts the colonization of pathogenic bacteria. It secretes proteases, facilitating the degradation of bacterial toxins and pathogen adhesion factors. Furthermore, through the activation of dendritic cells and toll-like receptors (TLR-2/4), this fungus enhances the intestinal secretion of IgA, regulates excessive inflammatory responses (Hedin et al., 2024), upregulates the expression of tight junction proteins, including occludin and ZO-1, and alleviates leaky gut syndrome. The soluble protein Trx, derived from *S. boulardii* exerted an array of significant effects on anti-inflammatory activity, including alleviating inflammation, protecting gut barrier, suppressing apoptosis, as well as reducing oxidative stress (Qin et al., 2024). Moreover, *S. boulardii* (CNCM I-745) plays a crucial role in the decomposition of SCFAs, providing essential energy for intestinal epithelial cells and participating in the complex regulation of bile acid metabolism (Yang et al., 2022). Currently, *S. boulardii* (CNCM I-745) has found widespread application in the treatment of pediatric diarrhea, inflammatory bowel disease, and other related conditions (Szajewska et al., 2020). *S. boulardii* (CNCM I-745) alleviates acetic acid-induced colitis in rats by regulating inflammation and barrier permeability (Altınok et al., 2025). Currently, *S. boulardii* (CNCM I-745), as a non-bacterial probiotic, is mostly used in the treatment of diarrhea and intestinal recovery after antibiotic treatment. This study specifically targeted rat pups as research subjects and endeavored to elucidate the mechanisms underlying the ameliorative effects of *S. boulardii* (CNCM I-745) on intestinal damage in sepsis. Our aim was to explore the potential impact of *S. boulardii* (CNCM I-745) on the long-term growth and development of children with sepsis, in order to provide new insights for the treatment of pediatric sepsis patients.

## 2 Materials and methods

### 2.1 Chemicals

*Saccharomyces boulardii* strain CNCM I-745 was acquired from Bioflor^®^CMS Shenzhen Kangzhe Pharmaceutical Co. Ltd. (Shenzhen, China) and manufactured by Biocodex (Paris, France). LPS was purchased from Sigma-Aldrich (St. Louis, USA). Serum IL-6 levels and TNF-α levels were measured using the commercial IL-6 ELISA Kit (Shanghai Enzyme-linked Biotechnology Co., Ltd., Rat, YJ102828) and TNF-α ELISA Kit (Shanghai Enzyme-linked Biotechnology Co., Ltd., Rat, YJ002859) according to the manufacturer’s instructions. All other materials used in the study were of analytical grade and obtained from reputable commercial suppliers.

### 2.2 Animals and treatment

The experimental protocols were previously approved by the Sixth Affiliated Hospital of Harbin Medical University (protocol number: LC2024-043). Forty-eight Wistar rats (4-5weeks), provided by Department of Experimental Animal Science, Harbin Medical University, weighing 80 g to 150 g, were used. All the rats mentioned were maintained in specific pathogen-free conditions and housed in cages, under controlled temperature (22-24°C) and humidity conditions, with 12 hours dark/light cycles, and maintained on standard diet with water throughout the experiment. Rats were randomly divided into four groups, n=12 in each group: group A: control group; group B: sepsis group; group C: probiotic group; group D: treatment group. Distilled water was administered orally by intragastric for 4 weeks in control group and sepsis group. *S. boulardii* (CNCM I-745) suspension (10^8^CFU/ml) was administered orally by intragastric for 4 weeks in probiotic group and treatment group. After establishing a new gut microbiota environment, we carried out intraperitoneal injections for each group of rats. Rats in control group and probiotic group were given intraperitoneal injection of normal saline (5ml/kg). Rats in sepsis group and treatment group were given intraperitoneal injection of LPS solution (1mg/ml, 5ml/kg). Half of the rats in each group (n=6) were euthanized after 8 hours of intraperitoneal injection, and the serum, colon tissue (2 cm distal to the ileocecal junction), and colon contents were collected and stored at -80 °C for further detection. 4 weeks later, the remaining rats (n=6) were sacrificed, and colonic contents samples were taken to observe the long-term changes of intestinal flora.

### 2.3 Histopathology

After fixation in 4% paraformaldehyde solution, the colonic tissue were taken for hematoxylin and eosin (H&E) to estimate colonic damage. Colon histological damage was assessed according to tissue damage and cell infiltration.

### 2.4 Immunohistochemistry

The RUNX2 (1:100), osteocalcin (1:100), goat anti-rabbit IgG antibody (1:200) and Occludin antibody (1:100) was acquired from Affinity Biosciences (Jiangsu, China). Colonic tissues were processed on formalin-fixed samples. The tissue sections embedded in paraffin were dewaxed, rehydrated, and antigen extracted on the basis of the procedure. The sections were stained with primary antibodies Occludin at 4°C, then turned to incubation with secondary antibody for fluorescent labeling. The finished were visualized by IHC using standard protocols. The intensity of chromogen staining in IHC was quantified by the ratio between the immunostaining area/total area (%) of the intestinal segment using Fiji software (ImageJ).

### 2.5 ELISA

For the determination of IL-6, and TNF-α concentrations, the abdominal aorta was punctured and the samples of blood were obtained. IL-6 and TNF-α levels were measured in serum by enzyme-linked immunosorbent assays using commercially available kits (ELISA).

### 2.6 RNA extraction, cDNA synthesis, and real time-quantitative PCR

The colonic tissues were swiftly frozen in liquid nitrogen and kept at -80t until extraction of total RNA using the guanidine thiocyanate-phenol-chloroform method. The RNA was dissolved in 20-50 μL of RNase-free water, and its concentration and purity were determined using a spectrophotometer (NanoDrop, Thermo Fisher Scientific, USA). First-strand cDNA was synthesized from 1 μg of total RNA. The reaction mixture included primers, reverse transcriptase, and dNTPs. The synthesized cDNA was stored at -20°C or used immediately for qPCR. The PCR conditions were 5 min at 94°C and 40 cycles of 10 s at 94°C, 30 s at 60°C for each amplification in a final volume of 20 µl. A melt curve analysis was performed at the end of the amplification to confirm the specificity of the PCR products. Each sample was run in triplicate to ensure reproducibility. The relative expression of specific genes in independent experiments was evaluated via 2−ΔΔCt method.

### 2.7 16S rRNA gene sequencing

The fecal microbiota profile was obtained through 16S rRNA sequencing analysis by Lianchuan Biotechnology Company. Microbial DNA was isolated from the intestinal contents of the rats to determine the purity of the DNA using 1% agarose gel electrophoresis. The bacterial ribosomal RNA gene V3–V4 sections were amplified, and specialized primers containing barcodes were produced. The AxyPrep DNA Gel Extraction Kit (Axygen Biosciences, Union City, CA, USA) was used to extract the PCR product from a 2% agarose gel and to purify it in accordance with the manufacturer’s instructions. We used the Illumina MiSeq PE300 platform (Illumina, San Diego, CA, USA) and followed industry standard procedures to pair-end sequence the purified amplicons.

### 2.8 Statistical analysis

GraphPad Prism version 8 (San Diego, CA, United States) was used to perform all analyses and prepare graphs. Drawing upon the specimens gathered at two distinct time points, the 4 primary groups (group A: control group; group B: sepsis group; group C: probiotic group; group D: treatment group) were meticulously subdivided into 8 subgroups (AW0, AW4, BW0, BW4, CW0, CW4, DW0, DW4). For all data displayed in graphs, the results are expressed as the mean ± SEM (n=6 per group). The differences in α-diversity indexes were analyzed by Student’s t-test. Plots of sample community compositions were compared using principal coordinate analysis (PCoA) and nonmetric multidimensional scaling (NMDS) according to weighted unifrac. The Anosim analysis is employed to assess whether the inter-group differences are significantly larger than the intra-group differences, thereby enabling the determination of whether the grouping strategy holds meaningful significance. For comparisons between two groups, Student’s t test for unpaired data was used. For comparisons among more than two groups, one-way analysis of variance (ANOVA) test were used. For all statistical tests, differences with a P value less than 0.05 were considered statistically significant: *P<0.05, **P<0.01, ***P<0.001,****P<0.0001.

## 3 Results

### 3.1 Sepsis leads to impaired intestinal barrier function

**Figure 1:**
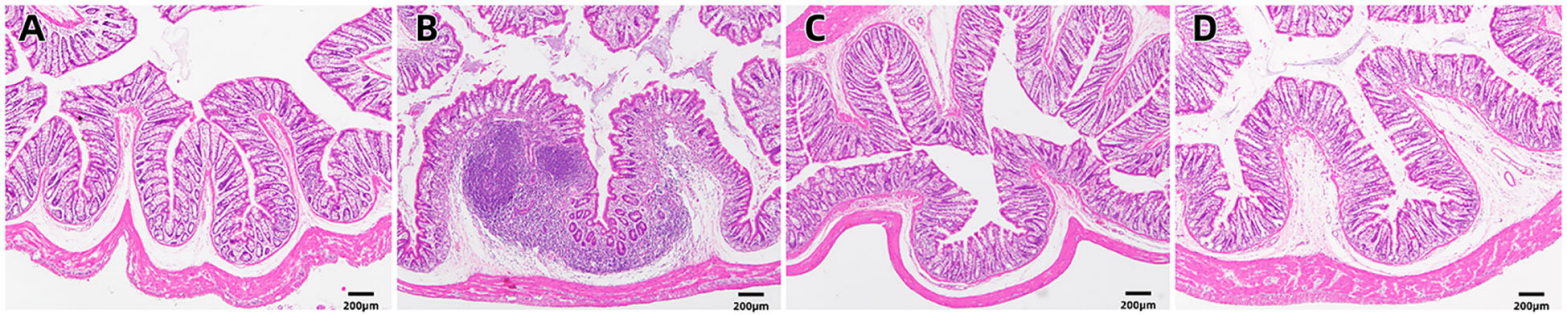
H&E staining of the intestinal mucosa in four groups [**(A)** control, **(B)** LPS, **(C)** *S. boulardii*, **(D)** *S. boulardii* + LPS, 40x].

**Figure 2:**
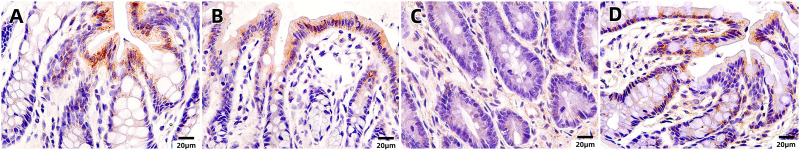
Immunohistochemical staining of the intestinal mucosa in four groups [**(A)** control, **(B)** LPS, **(C)** *S. boulardii*, **(D)** *S. boulardii* + LPS, 400x].

**Figure 3:**
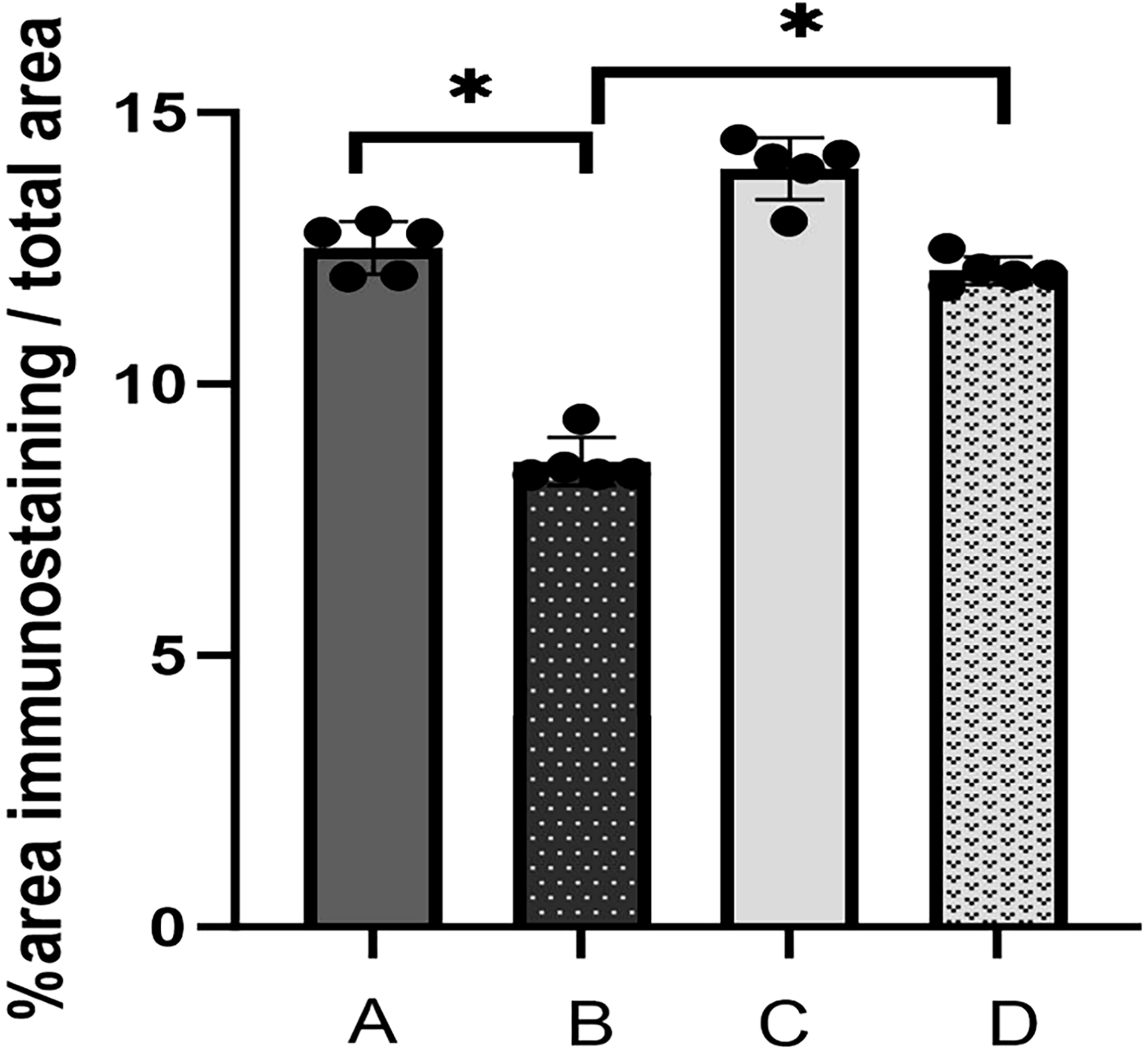
The immunohistochemical index of occludin was determined semiquantitatively in the proximal colonic tissue. Values in Figure 3 are presented as mean ± SEM % of the ratio of the area of immunostaining/total area (A: control, B: LPS, C: *S. boulardii*, D: *S. boulardii* + LPS, *P<0.05, ordinary one-way ANOVA).

Upon examination of the histopathological findings (**Figure 1**), the intestinal tissue of the control group exhibited normal intestinal mucosal villi. Conversely, in the sepsis group, there was an enlargement of intestinal mucosal gaps, congestion of capillaries, and a pronounced infiltration of inflammatory cells within the intestinal tissue. In contrast, the probiotic group displayed normal intestinal mucosal cells. The treatment group demonstrated a limited infiltration of inflammatory cells in the intestine and exhibited a marked reduction in intestinal damage when compared to the sepsis group. In light of these histological alterations, immunohistochemical analysis was conducted to gain further insights into the functional changes of the intestinal mucosal barrier. Immunohistochemical staining revealed that the expression levels of occludin were notably decreased in the sepsis group compared to the control group (*P<0.05). Notably, the treatment group exhibited significantly elevated expression of occludin in comparison to the sepsis group (*P<0.05, **Figures 2**, **3**).

### 3.2 *S. boulardii* (CNCM I-745) improves intestinal injury in sepsis

**Figure 4:**
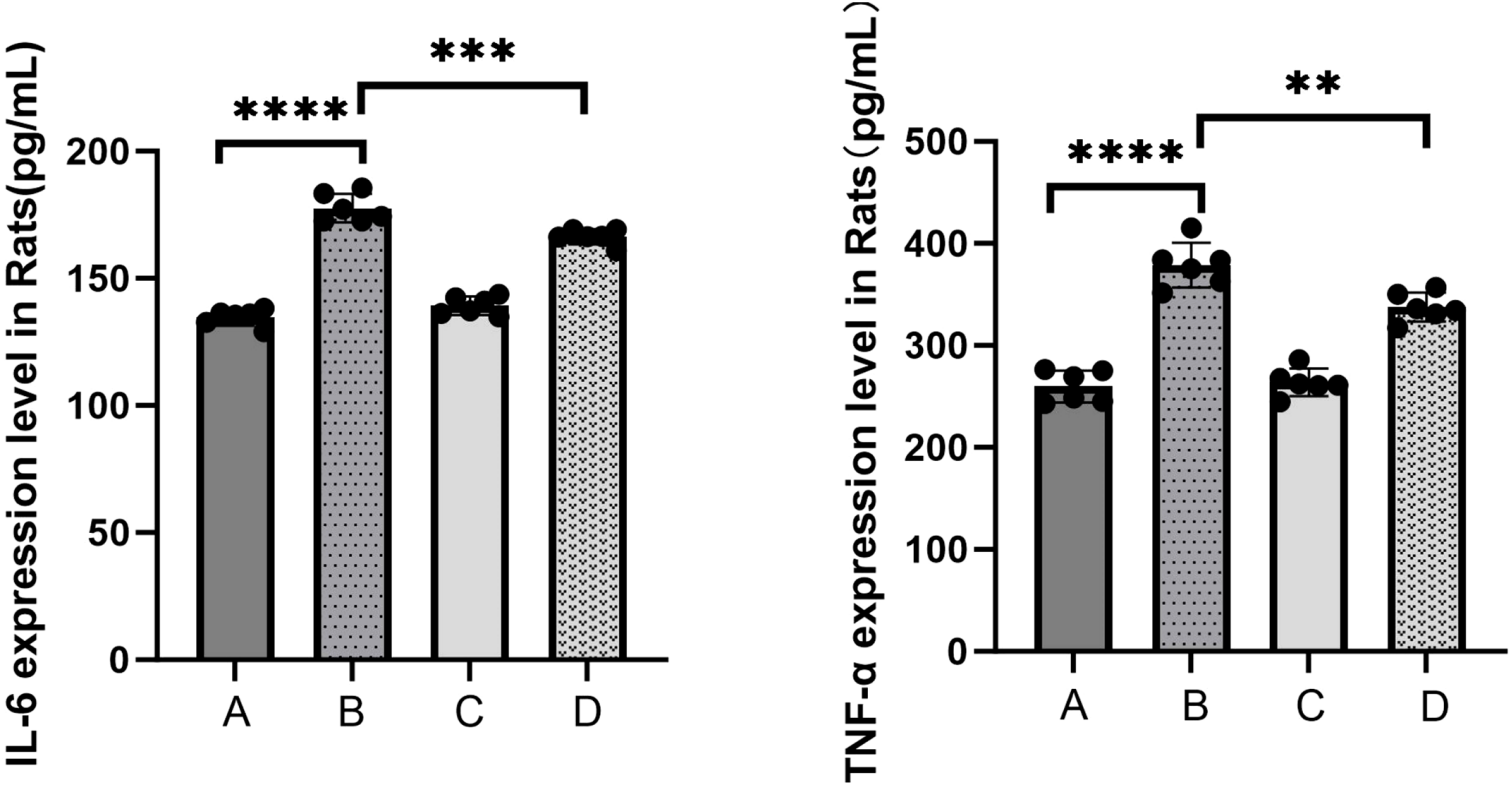
The levels of serum inflammatory mediators IL-6 and TNF-α were detected by ELISA kits (A: control, B: LPS, C: *S. boulardi* ***i***, D: *S. boulardii* + LPS, **P<0.01, ***P<0.001,****P<0.0001, ordinary one-way ANOVA).

**Figure 5:**
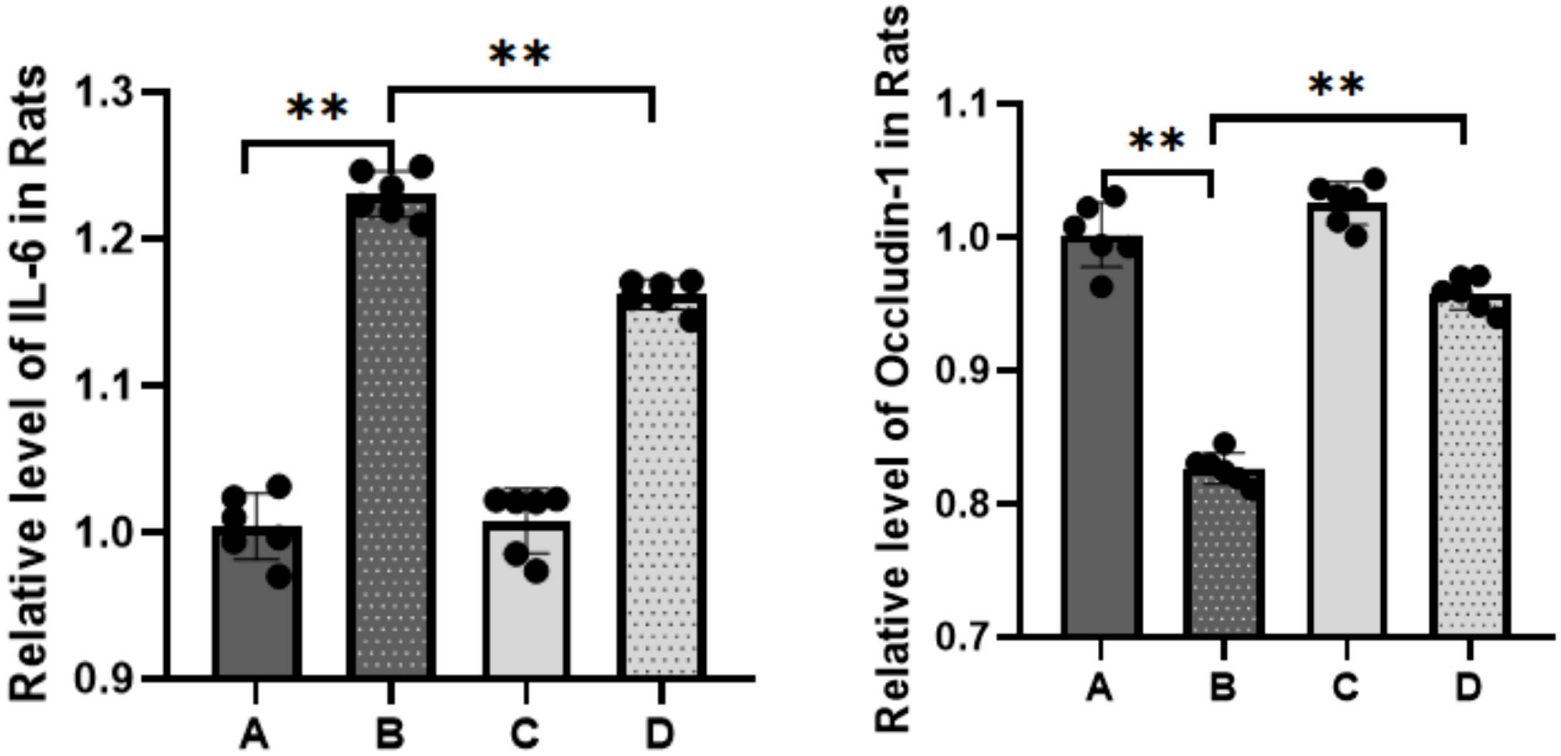
Expression of IL-6 and occludin detected by RT-qPCR (A: control, B: LPS, C: *S. boulardii*, D: *S. boulardii* + LPS, **P<0.01, ordinary one-way ANOVA).

To elucidate the inflammatory responses in each rat group, ELISA assays were performed using blood samples (**Figure 4**). The results indicated a substantial increase in the expression of inflammatory factors in the sepsis group compared to the control group (****P<0.0001). In contrast, the treatment group exhibited a statistically significant decrease in the expression levels of IL-6 (***P<0.001) and TNF-α (**P<0.01) compared to the sepsis group. The qPCR results (**Figure 5**) demonstrated that, in comparison to the control group, the sepsis group exhibited a significant increase in the expression of inflammatory factors, accompanied by a notable decrease in the expression of occludin (**P<0.01). Furthermore, the treatment group showed a significant reduction in the expression of inflammatory factors and a significant increase in the expression of occludin when compared to the sepsis group (**P<0.01).

These findings suggest that sepsis impairs intestinal barrier function and exacerbates inflammatory responses. Importantly, *S. boulardii* (CNCM I-745) treatment mitigates intestinal inflammatory damage in sepsis and positively contributes to the maintenance of intestinal barrier function.

### 3.3 *S. boulardii* (CNCM I-745) alters the composition of gut microbiota in sepsis induced intestinal injury

**Figure 6:**
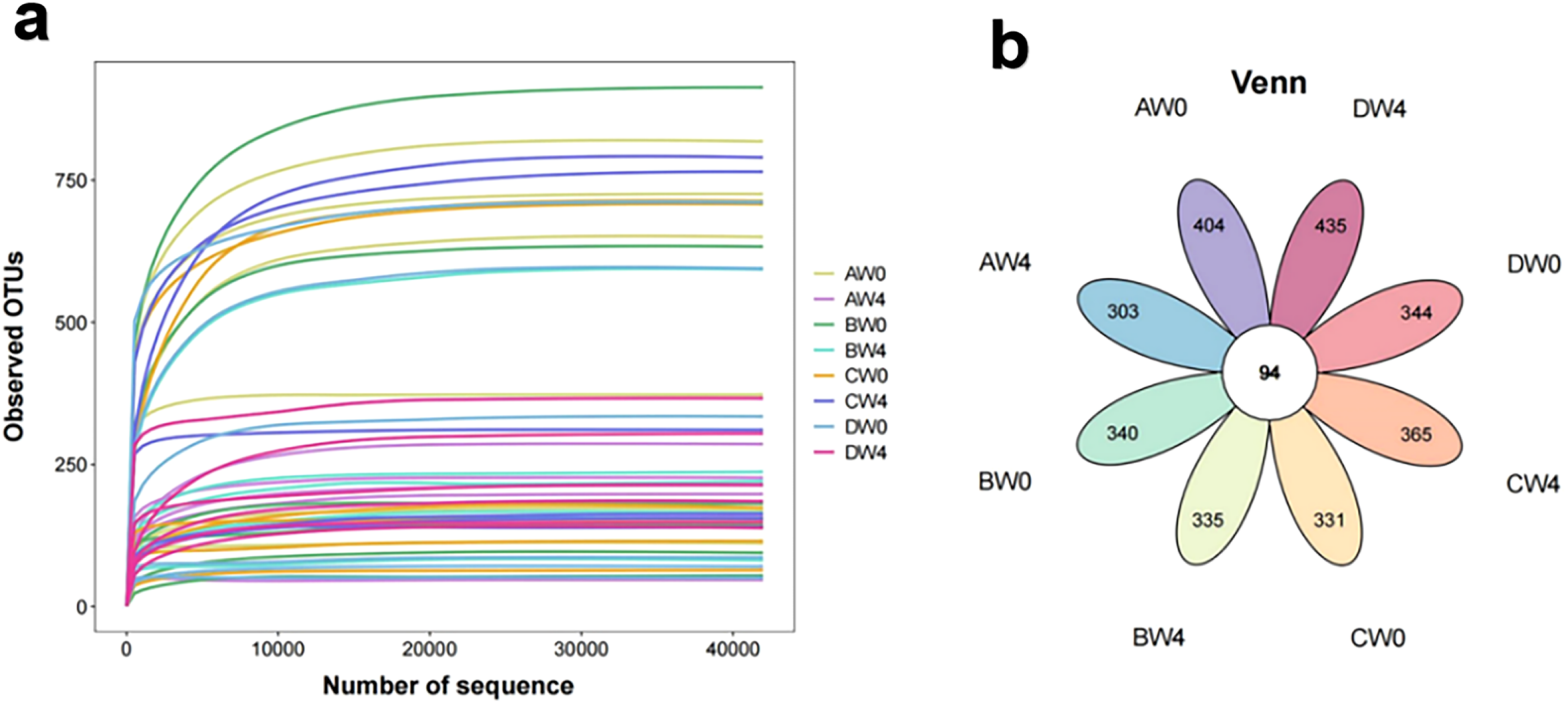
**(a)** The rarefaction curve of the observed OTUs. **(b)** A Venn diagram showing the overlap of the OTUs identified in the intestinal microbiota among eight groups (AW0, control group taken 8 hours after intraperitoneal injection; BW0, sepsis group taken 8 hours after intraperitoneal injection; CW0, probiotic group taken 8 hours after intraperitoneal injection; DW0, treatment group taken 8 hours after intraperitoneal injection; AW4, control group taken 4 weeks after intraperitoneal injection; BW4, sepsis group taken 4 weeks after intraperitoneal injection; CW4, probiotic group taken 4 weeks after intraperitoneal injection; DW4, treatment group taken 4 weeks after intraperitoneal injection).

The index dilution curve plateaued with increasing data extraction volume, suggesting appropriate sequencing data volume. The Venn diagram showing the overlap of the OTUs identified in the intestinal microbiota among four groups (**Figure 6**).

**Figure 7:**
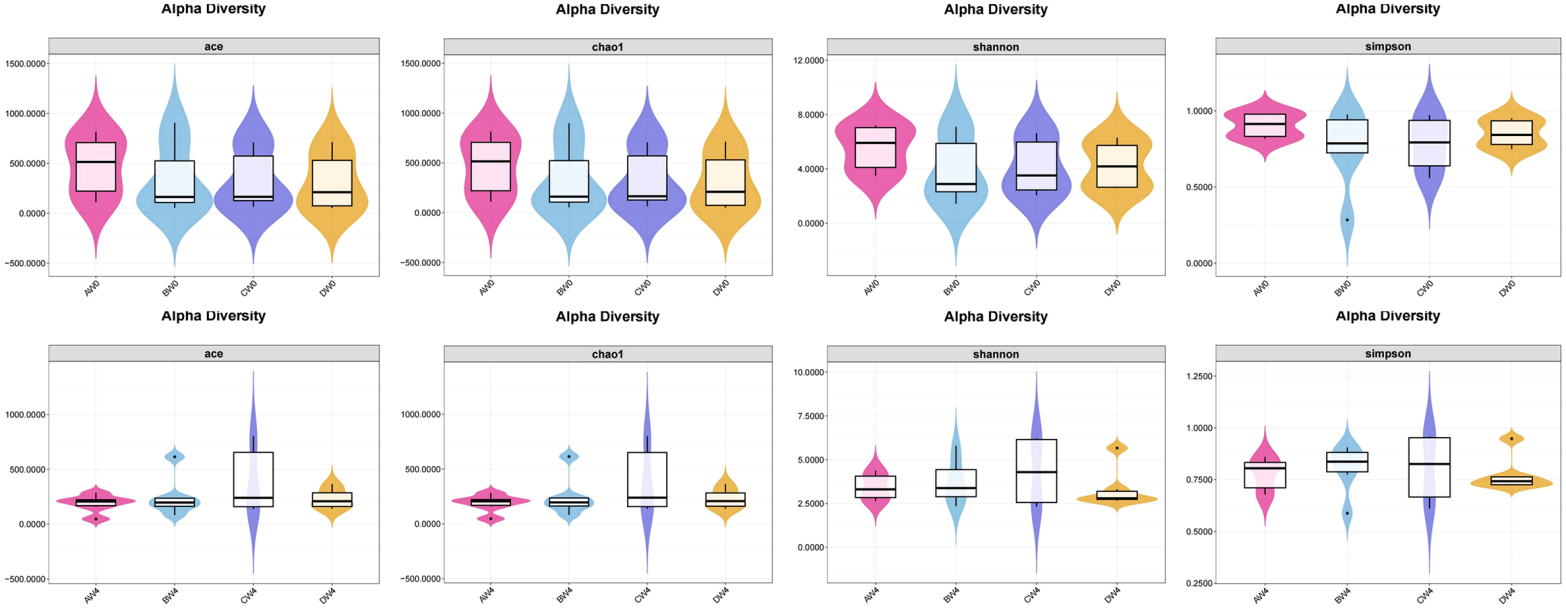
The α-diversity indexes of gut microbiota among the groups (*P>*0.05).

Alpha diversity analysis (**Figure 7**). To assess species abundance, we utilized the ACE index and Chao1 index, whereas microbial diversity was determined through a comparative analysis of the Shannon index and Simpson index. Following intraperitoneal injection, alpha diversity results revealed that eight hours post-injection, the control group exhibited higher ACE index, Chao1 index, Shannon index, and Simpson index values compared to the other three groups. Notably, the treatment group demonstrated superior abundance and diversity of intestinal microbiota relative to the sepsis group. Four weeks subsequent to intraperitoneal injection, we observed the abundance and diversity of gut microbiota across all groups. The probiotic group displayed enhanced gut microbiota abundance and diversity compared to the remaining three groups. However, these differences did not reach statistical significance among the groups (*P>*0.05).

**Figure 8:**
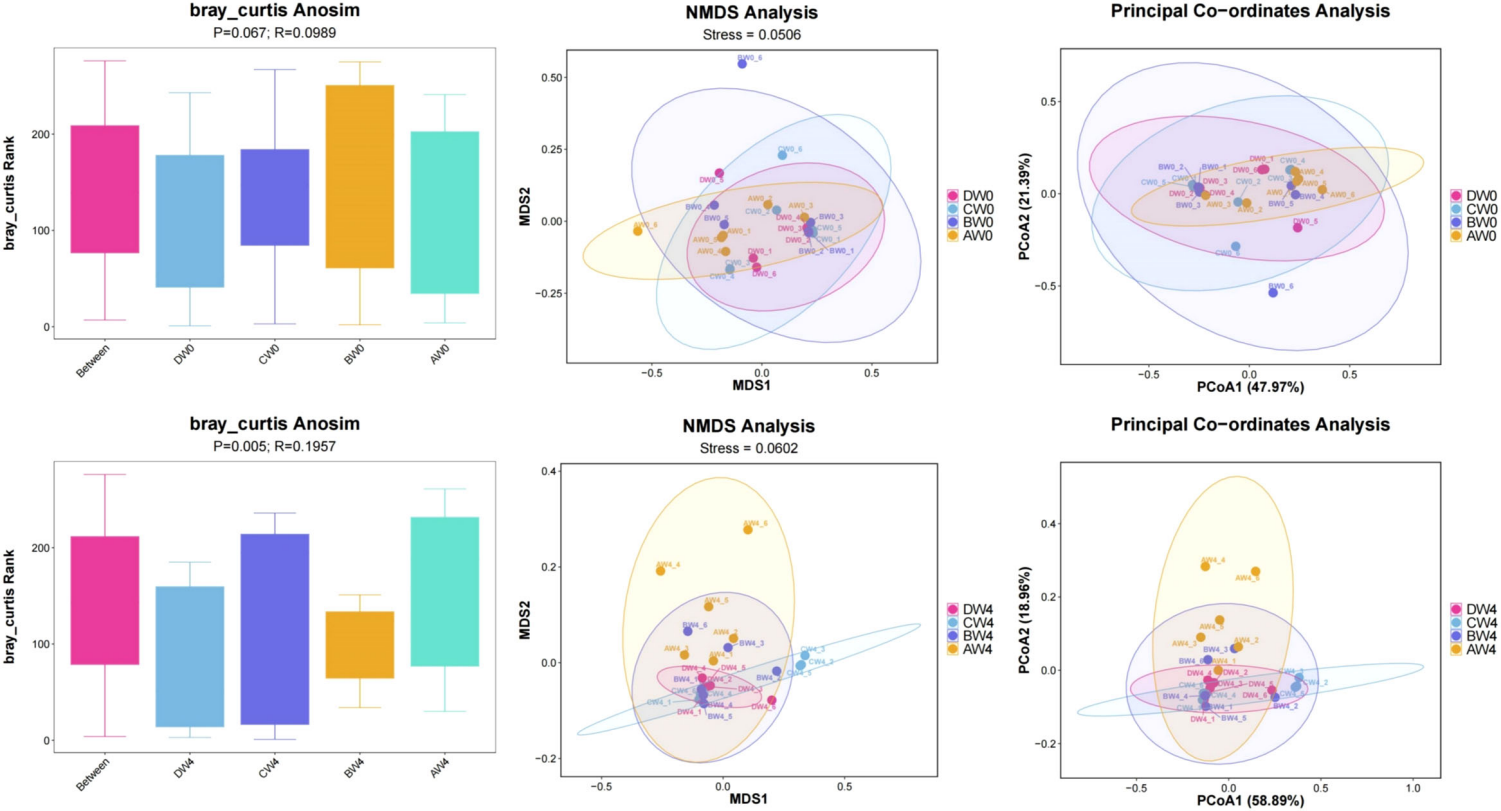
The β-diversity indexes of gut microbiota among the groups.

Beta diversity analysis (**Figure 8**). To examine the variations among different groups, the Anosim test was employed. Following the analysis of samples collected 8 hours post-intraperitoneal injection, the researchers observed notably greater disparities in community composition among the four groups-comprising the control group, sepsis group, probiotic group, and treatment group-compared to the variations within each individual group (R=0.0989, P=0.067). These groups were further subjected to Non-metric Multidimensional Scaling (NMDS) analysis and Principal Coordinate Analysis (PCoA). Upon analyzing samples obtained 4 weeks post-intraperitoneal injection, we found even more pronounced differences in community composition among the four groups, relative to the differences within each group (R=0.1957, P=0.005). By considering all available sequences in a comparative analysis, a strikingly high level of β-diversity (heterogeneity) was revealed among the groups.

**Figure 9:**
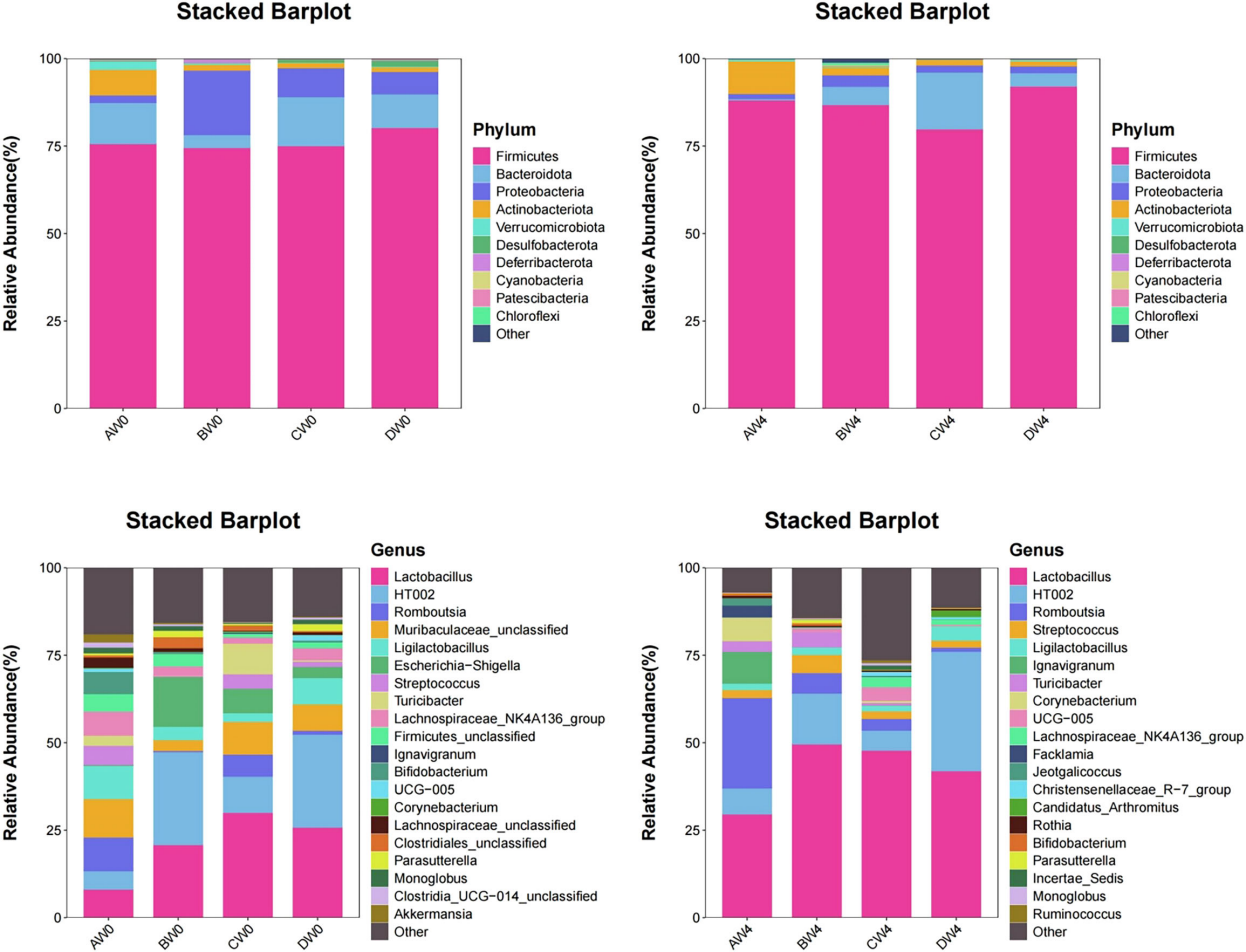
The proportion of microbial composition among the groups at the phylum and genus levels.

Species composition analysis (**Figure 9**). At the phylum level, the highest proportions are observed for *Firmicutes*, *Bacteroidetes*, *Proteobacteria*, and *Actinobacteria*. On the initial day, the *Firmicutes*-to-*Bacteroidetes* ratio was notably lowest in the probiotic group, whereas it was highest in the sepsis group. Four weeks post-initiation, the probiotic group maintained the lowest *Firmicutes*-to-*Bacteroidetes* ratio. At the genus level, on the first day, the sepsis group exhibited the highest abundance of *Escherichia-Shigella* and *Corynebacterium*. Conversely, the probiotic group demonstrated the highest abundance of *Lactobacillus* and *Turicibacter*. The control and treatment groups showed higher abundance of *Romboutsia*. Four weeks later, the sepsis group had the highest abundance of *Streptococcus*. Notably, the proportion of *Lactobacilli* in each group increased compared to their levels four weeks prior.

**Figure 10:**
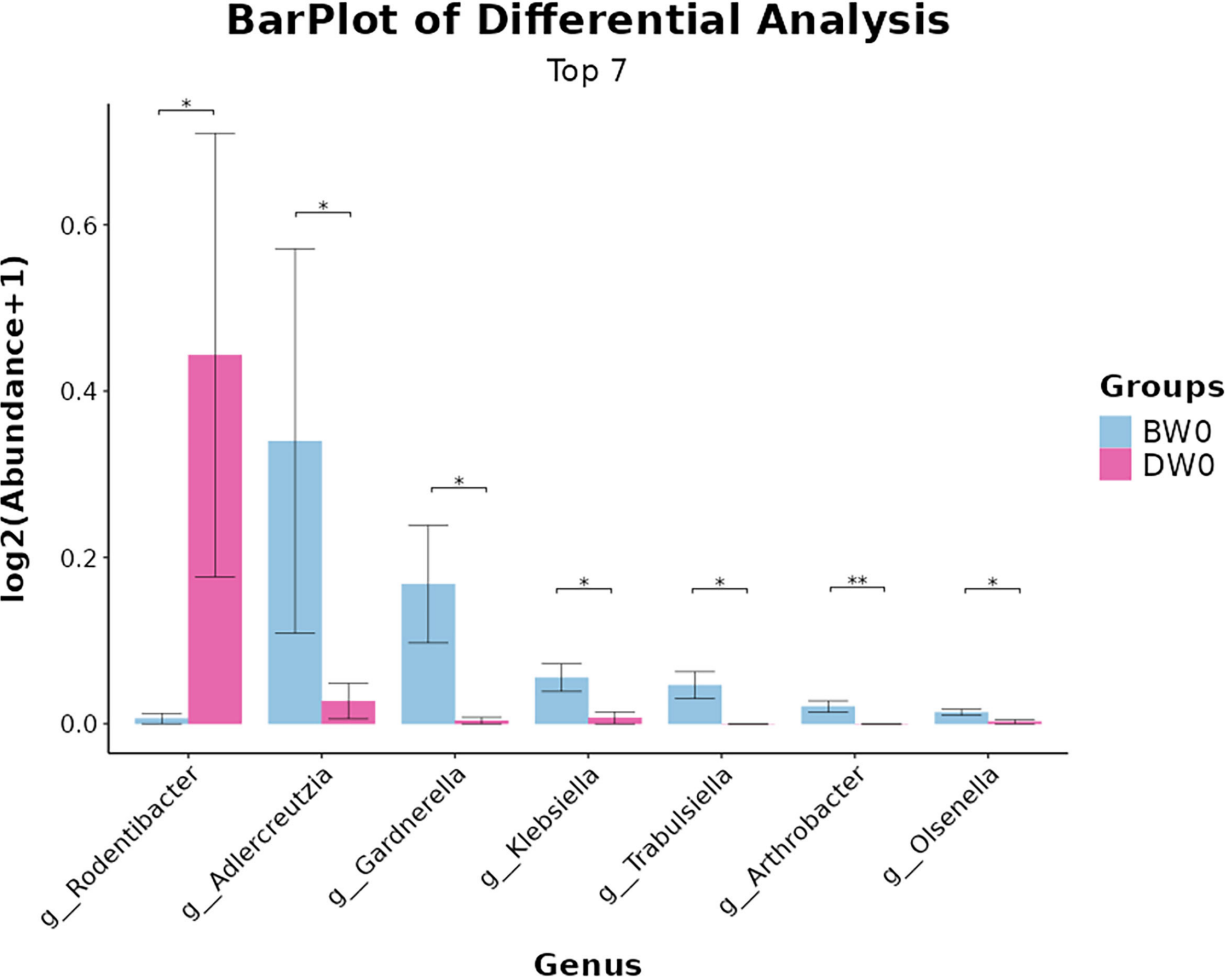
Differences in microbial composition among the groups at the genus levels (*P<0.05, **P<0.01).

Species difference analysis (**Figure 10**). We conducted a comparative analysis of the top seven microbial communities at the genus level, which exhibited statistically significant differences between the sepsis and treatment groups. The abundance of *Rodentibacter* was significantly higher in the treatment group compared to the sepsis group. Conversely, the sepsis group exhibited significantly higher abundances of *Adlercreutzia*, *Gardnerella*, *Klebsiella*, *Trabulsiela*, *Arthrobacter*, *Olsenella*, and notably lower abundances of *Bifidobacterium* compared to the treatment group.

**Figure 11:**
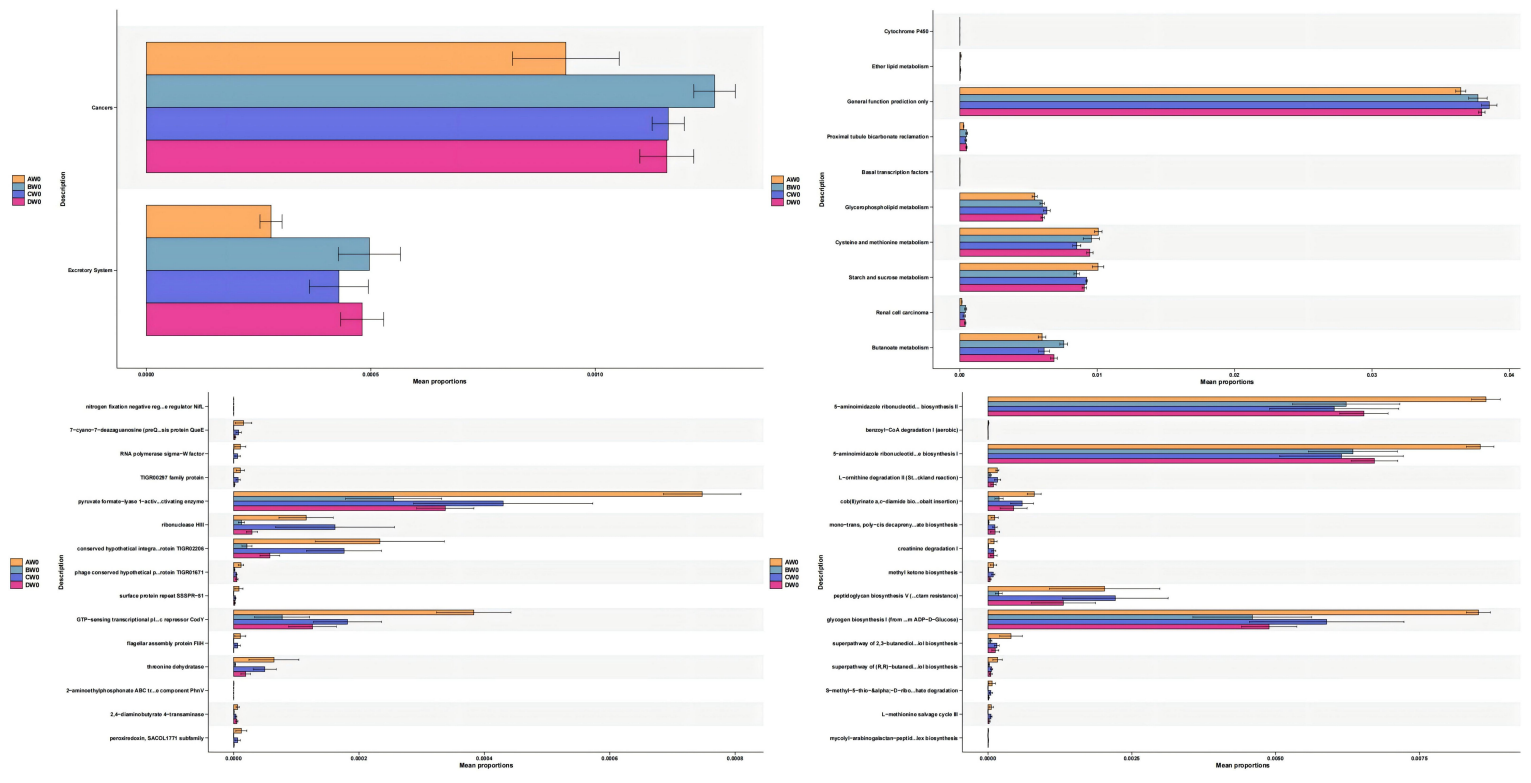
Changes in various metabolic pathways among the groups.

Functional Predictive Analysis (**Figure 11**). We employed PICRUSt2 to forecast the functional composition of microbial communities within the amplicon sequencing samples. Our findings revealed that the intestinal flora subsequent to sepsis-induced intestinal injury impacts a diverse array of metabolic pathways, including cysteine and methionine metabolism, as well as starch and sucrose metabolism. The administration of *S. boulardii* (CNCM I-745) mitigated these effects. Intriguingly, we observed that alterations in the gut microbiota of sepsis patients resulted in an upregulation of metabolic pathways associated with cancer and the excretory system. However, the expression of these metabolic pathways was downregulated following the application of *S. boulardii* (CNCM I-745).

## 4 Discussion

It was well established that a robust intestinal mucosal barrier and a stable intestinal microbiota structure were pivotal for maintaining intestinal health (Gasaly et al., 2021 and An et al., 2022). A healthy gut is characterized by a higher abundance and greater diversity of beneficial bacteria (Wilson et al., 2024 and Marsaux et al., 2023). We administered a solution of *S. boulardii* to rats via continuous gavage to establish a new balance in their intestinal environment. When the rats were exposed to LPS - induced injury, those that had received prophylactic treatment with *S. boulardii* displayed markedly less intestinal inflammatory infiltration compared to the sepsis group. Additionally, the expression levels of intestinal tight - junction proteins in the treated rats were higher than those observed in the sepsis group. High - throughput sequencing results revealed that the administration of *S. boulardii* led to an increase in the diversity and abundance of the gut microbiota. It also altered the microbiota’s structure, promoting the colonization of beneficial bacteria while curbing that of harmful ones. Based on these findings, we hypothesize that the novel gut microbiota environment established through prophylactic *S. boulardii* treatment can strengthen the intestinal barrier, modulate immune responses, and decrease susceptibility to sepsis. However, the precise underlying mechanisms remain elusive. Previous studies have shown that *S. boulardii* (CNCM I-745) may partially maintain intestinal mucosal integrity through TLR2/MYD88/NF-κB pathway inhibition (Ahmad et al., 2024). *S. boulardi*i (CNCM I-745) restores intestinal barrier Integrity by regulation of E-cadherin recycling administration of *S. boulardi*i (CNCM I-745) mitigated intestinal damage by augmenting the expression of tight junction proteins and decreasing the secretion of inflammatory mediators (Terciolo et al., 2017). Previous studies have found *Firmicutes* specialize in breaking down complex carbohydrates to produce SCFAs, which promote energy absorption (Agus et al., 2021). *Bacteroidetes*, on the other hand, decompose dietary fiber and host polysaccharides, maintaining intestinal barrier function (Ross et al., 2024 and Li et al., 2024).

From the analysis of species composition, we found that the use of *S. boulardii* (CNCM I-745) in sepsis treatment reduced the ratio of *Firmicutes* to *Bacteroidetes* in the gut microbiota. It also led to decreased colonization of harmful bacteria such as *Escherichia-Shigella* and *Corynebacterium*, while increasing the colonization of *Lactobacillus*, *Turicibacter*, and other microbiota. Studies have demonstrated that the ratio of *Firmicutes* to *Bacteroidetes* typically maintains a dynamic equilibrium, with changes closely associated with human health, metabolic diseases, and nutritional status (Hills et al., 2019). An increased ratio is commonly observed in obesity, diabetes, and metabolic syndrome, whereas a decreased ratio is linked to emaciation, malnutrition, or specific intestinal inflammatory diseases such as ulcerative colitis (Guo et al., 2023 and Xie et al., 2023 and Mokhtari et al., 2021). The administration of *S. boulardii* (CNCM I-745) may exert an influence on the long-term growth and development of pediatric sepsis patients (Healy et al., 2022). However, recent research has highlighted significant individual variations in this ratio, making it challenging to establish a unified standard. Therefore, this ratio cannot serve as a definitive “barometer” of gut microbiota health. Previous investigations have established that *Turicibacter* strains possess the capability to modulate genes implicated in host bile acid and lipid metabolism, thereby eliciting reductions in serum cholesterol levels, triglycerides, and adipose tissue mass (Lynch et al., 2023). Currently, the bulk of research endeavors on *Turicibacter* primarily rely on animal models, leaving the precise role it plays in the human gut microbiome largely unelucidated.

Our study conducted an analysis of species variations and revealed that the administration of *S. boulardii (*CNCM I-745) in the context of sepsis led to a decrease in the colonization of *Gardnerella*, *Klebsiella*, *Trabulsiela*, and other microbial communities. *Gardnerella* is capable of secreting sialidase and mucin, which can disrupt the host’s mucus barrier and facilitate the establishment of other pathogenic bacteria (Castro et al., 2019 and Anton et al., 2022). *Klebsiella*, an important opportunistic pathogen within the *Enterobacteriaceae* family, promotes host cell adhesion and biofilm formation, thereby enhancing bacterial persistence within the host and triggering systemic inflammatory responses (Watanabe et al., 2024 and da Silva Bandeira et al., 2025). Based on these findings, we speculated that the reduction of these harmful bacteria might be related to the alleviation of intestinal damage and could potentially influence the long - term prognosis. The functional prediction outcomes of this study suggest that the utilization of *S. boulardii (CN*CM I-745) in the management of sepsis notably diminishes the expression of metabolic pathways associated with cancer and the excretory system. Previous research has elucidated that this synthetically engineered probiotic yeast has the capacity to engage with the PD-1/PD-L1 pathway, thereby alleviating intestinal tumor burden in a mouse model of colorectal cancer that is refractory to standard immune checkpoint inhibitor (ICI) therapy (Wong and Yu, 2023). This tailored therapeutic approach holds promise for innovating treatment strategies for gastrointestinal malignancies. Beyond its application in sepsis treatment, *S. boulardii* (CNCM I-745) is anticipated to assume an unforeseen, yet promising, role in the management of cancer and digestive system disorders.

Additionally, it is imperative to exercise caution regarding the dosage and timing of administering probiotics like *S. boulardii (C*NCM I-745). Improper usage may inadvertently predispose individuals to fungal infections. Timely prevention, early diagnosis, and effective intervention in sepsis are pivotal for enhancing clinical outcomes. To this end, augmenting global awareness of sepsis, elevating individual educational standards, and refining public health policies are indispensable. Given the intricate and heterogeneous nature of sepsis pathogenesis and treatment, the incorporation of probiotics stands poised to significantly contribute to the refinement of personalized and diversified therapeutic paradigms. As technological advancements continue to unfold, artificial intelligence may hold the potential to revolutionize research in sepsis and gut microbiota, offering unprecedented insights and solutions.

## 5 Conclusion

Collectively, sepsis induces impairment of intestinal barrier function and exacerbates inflammatory responses. The administration of *S. boulardii (CN*CM I-745) can modify the composition of the intestinal microbiota, subsequently mitigating inflammatory damage and aiding in the preservation of the intestinal mucosal barrier’s functionality. The new intestinal environment established following the use of *S. boulardii (CN*CM I-745) might influence the long-term growth and development of pediatric patients suffering from sepsis. These discoveries offer a fresh perspective for the treatment of pediatric sepsis. However, additional research is imperative to elucidate the precise mechanisms through which these dominant microbiota and their metabolites exert the effects.

## Data Availability

The original contributions presented in the study are included in the article/**Supplementary Material**. Further inquiries can be directed to the corresponding author/s.
