# Supplementary figures and images for: Saccharomyces boulardii (CNCM I-745) improves intestinal damage in sepsis by remodeling intestinal flora structure
Source: Front Cell Infect Microbiol. 2025 Jul 31;15:1602792. doi: 10.3389/fcimb.2025.1602792 (PMC12350329; doi:10.3389/fcimb.2025.1602792)

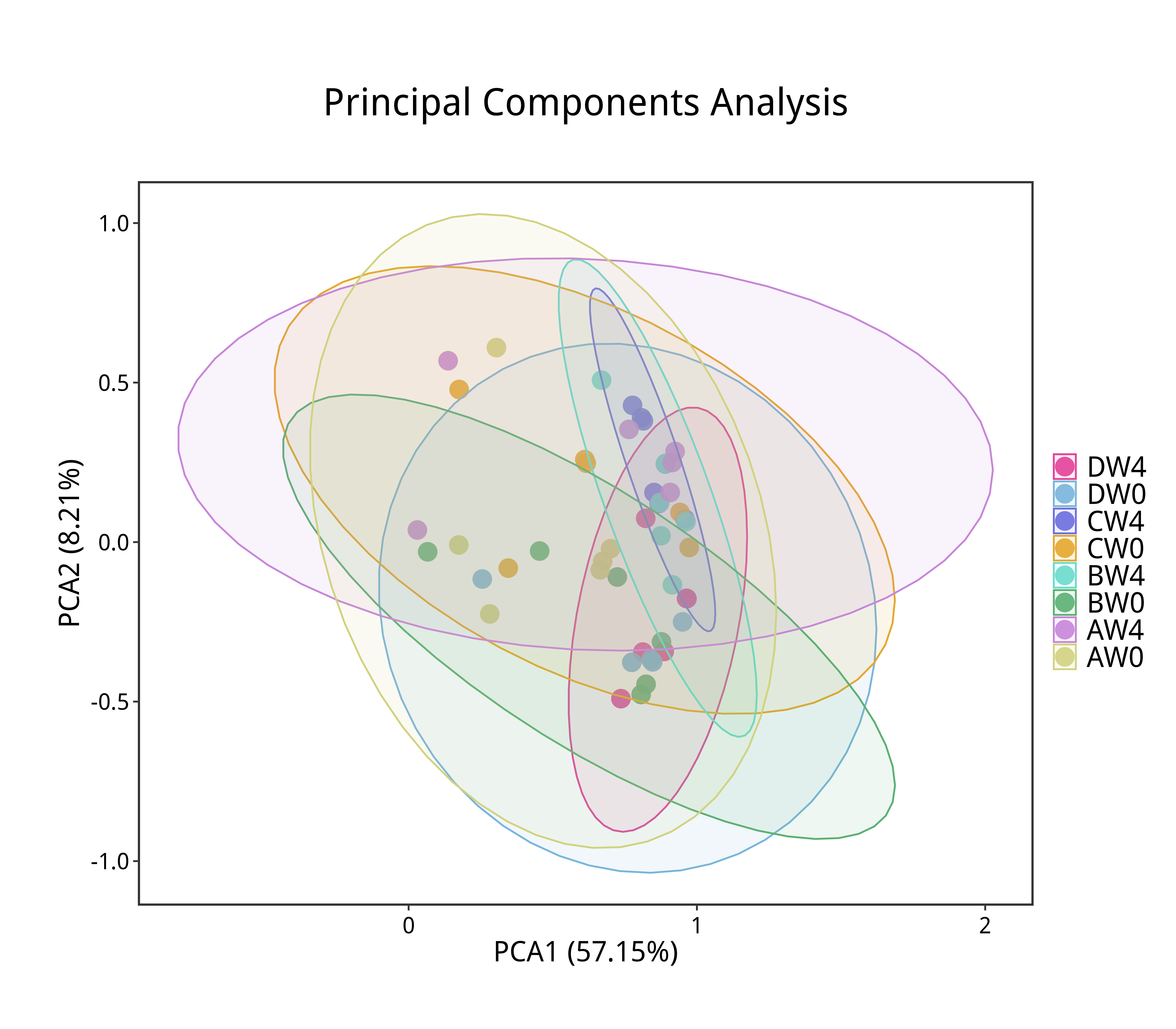

Supplement: Supplementary file 1 [file DataSheet1.zip › summary/summary/5_Beta_diversity/2_PCA_result/Beta_Diversity.PCA.no_label.png]

# Principal Components Analysis

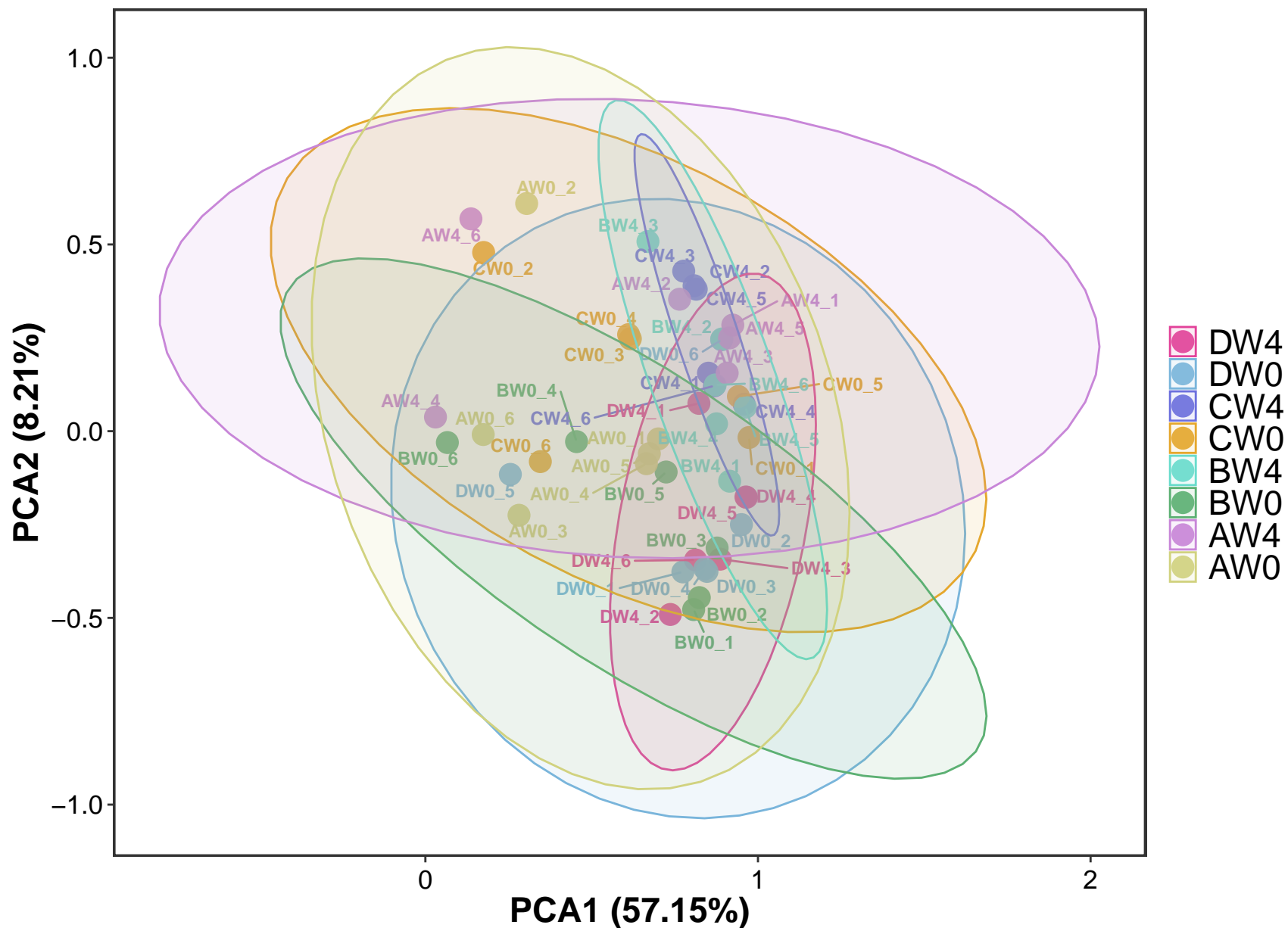

Supplement: Supplementary file 1 [file DataSheet1.zip › summary/summary/5_Beta_diversity/2_PCA_result/Beta_Diversity.PCA.with_label.pdf]

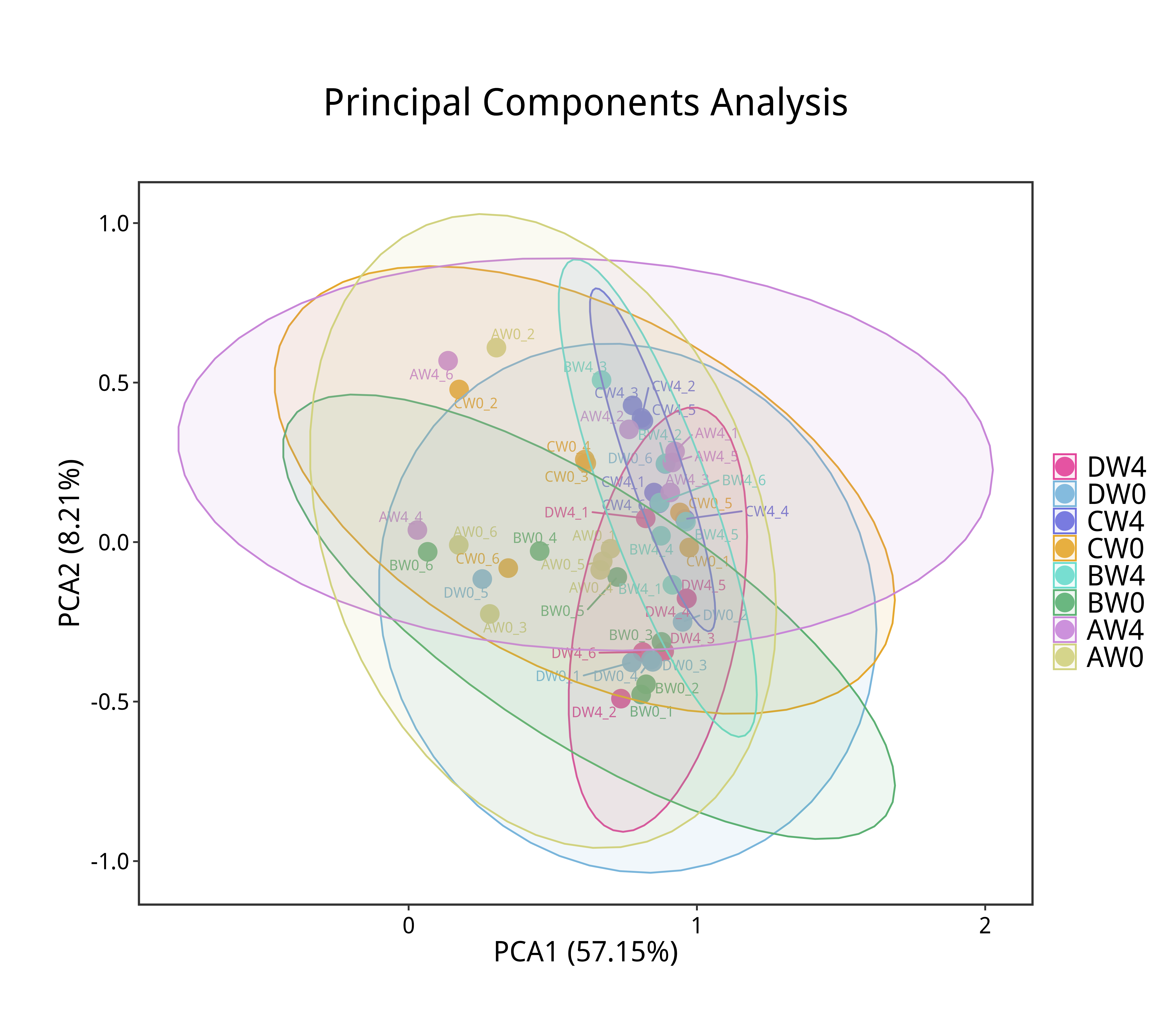

Supplement: Supplementary file 1 [file DataSheet1.zip › summary/summary/5_Beta_diversity/2_PCA_result/Beta_Diversity.PCA.with_label.png]

# Principal Components Analysis

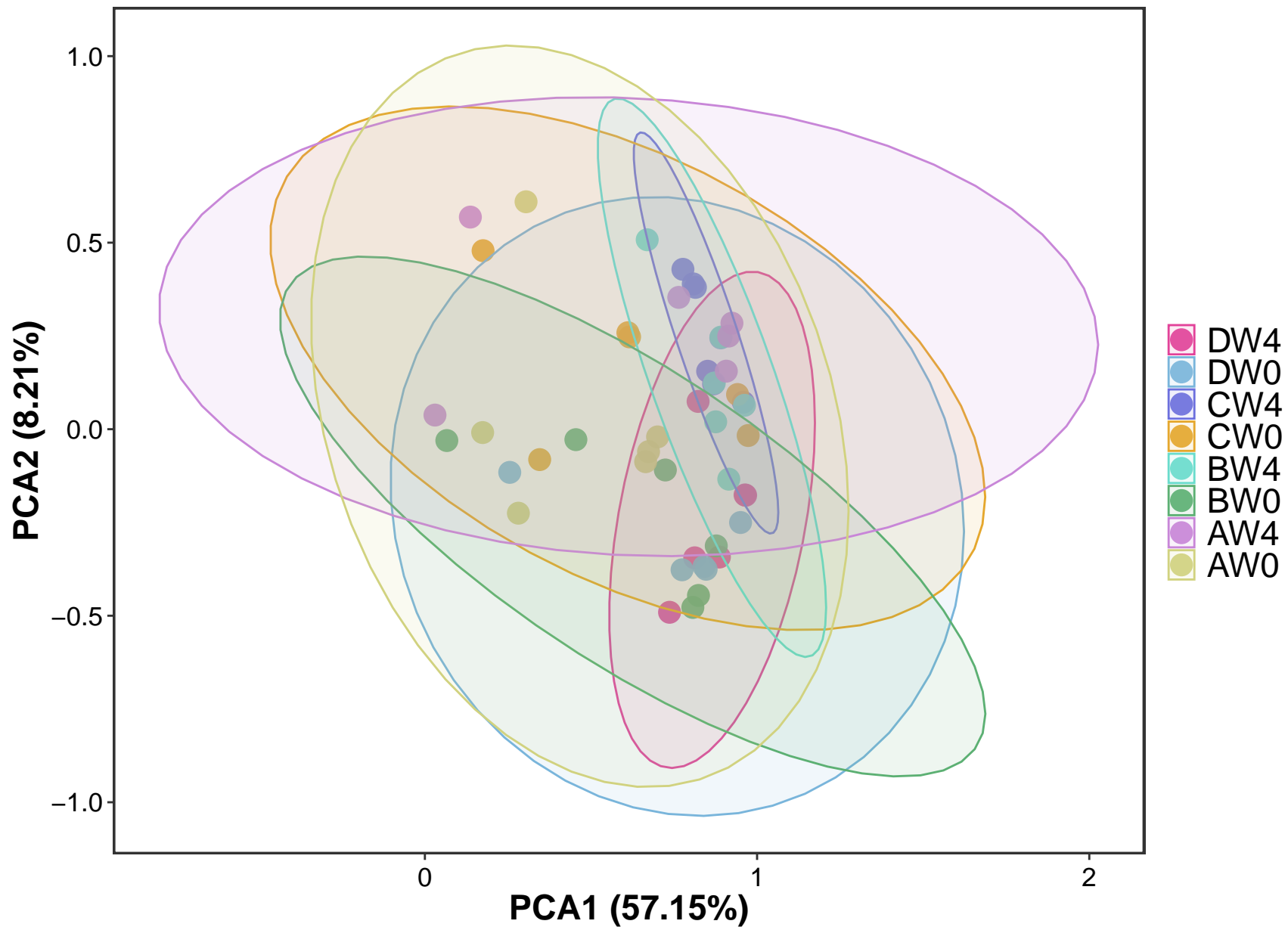

Supplement: Supplementary file 1 [file DataSheet1.zip › summary/summary/5_Beta_diversity/2_PCA_result/Beta_Diversity.PCA.no_label.pdf]

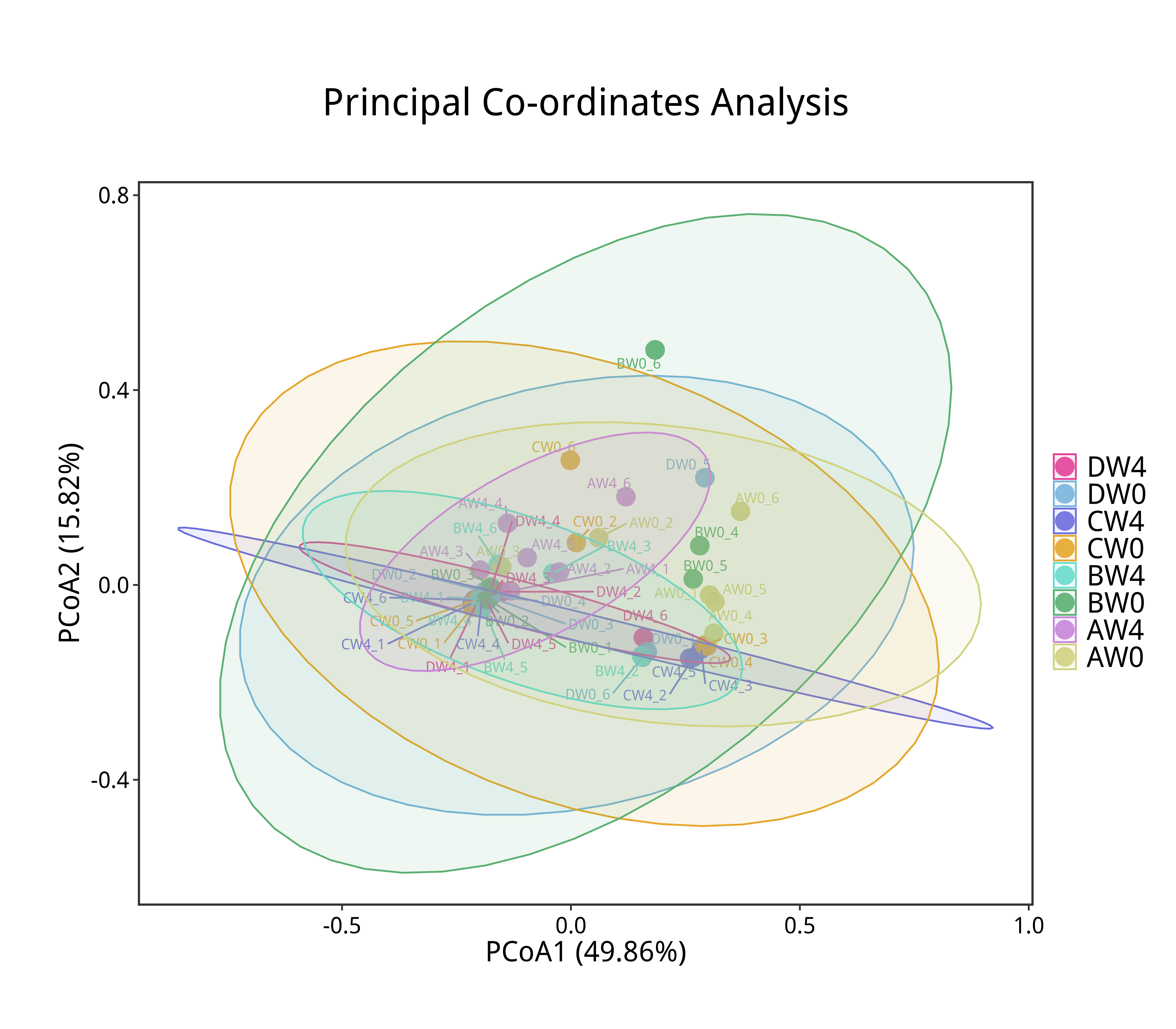

Supplement: Supplementary file 1 [file DataSheet1.zip › summary/summary/5_Beta_diversity/3_PCoA_result/PCoA_2D/Beta_Diversity.weighted_unifrac.PCoA.with_label.png]

# Principal Co-ordinates Analysis

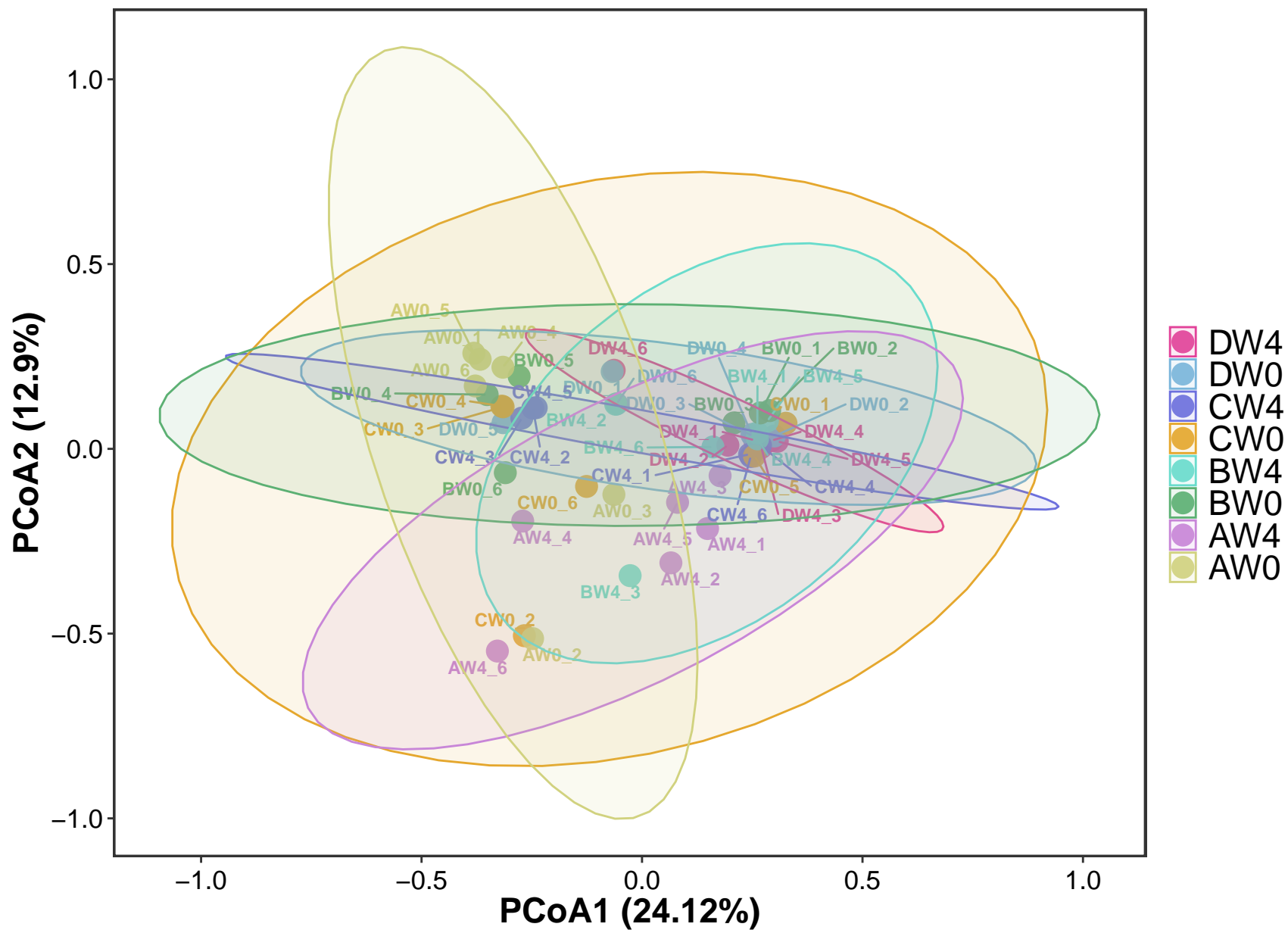

Supplement: Supplementary file 1 [file DataSheet1.zip › summary/summary/5_Beta_diversity/3_PCoA_result/PCoA_2D/Beta_Diversity.bray_curtis.PCoA.with_label.pdf]

# Principal Co-ordinates Analysis

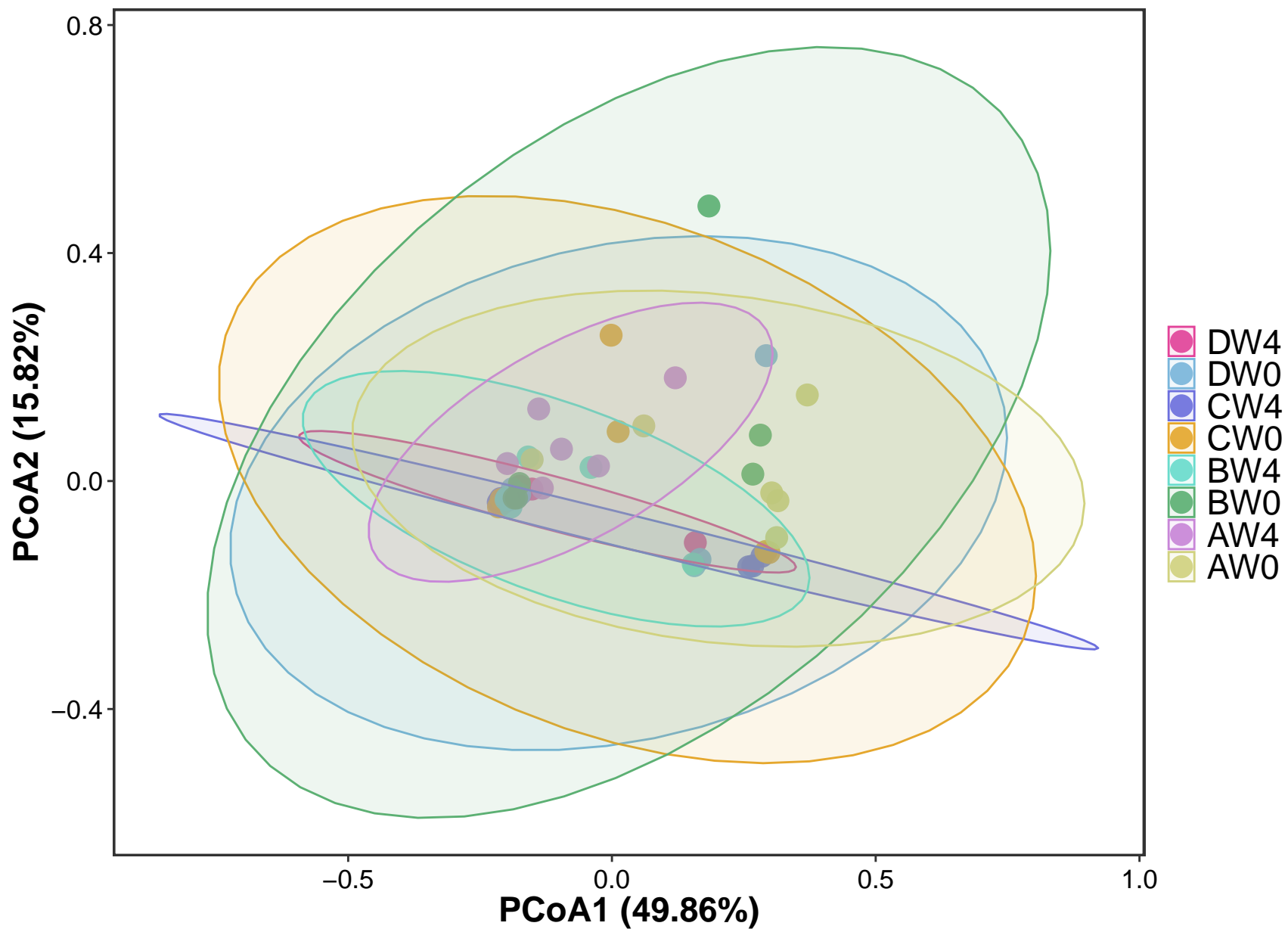

Supplement: Supplementary file 1 [file DataSheet1.zip › summary/summary/5_Beta_diversity/3_PCoA_result/PCoA_2D/Beta_Diversity.weighted_unifrac.PCoA.no_label.pdf]

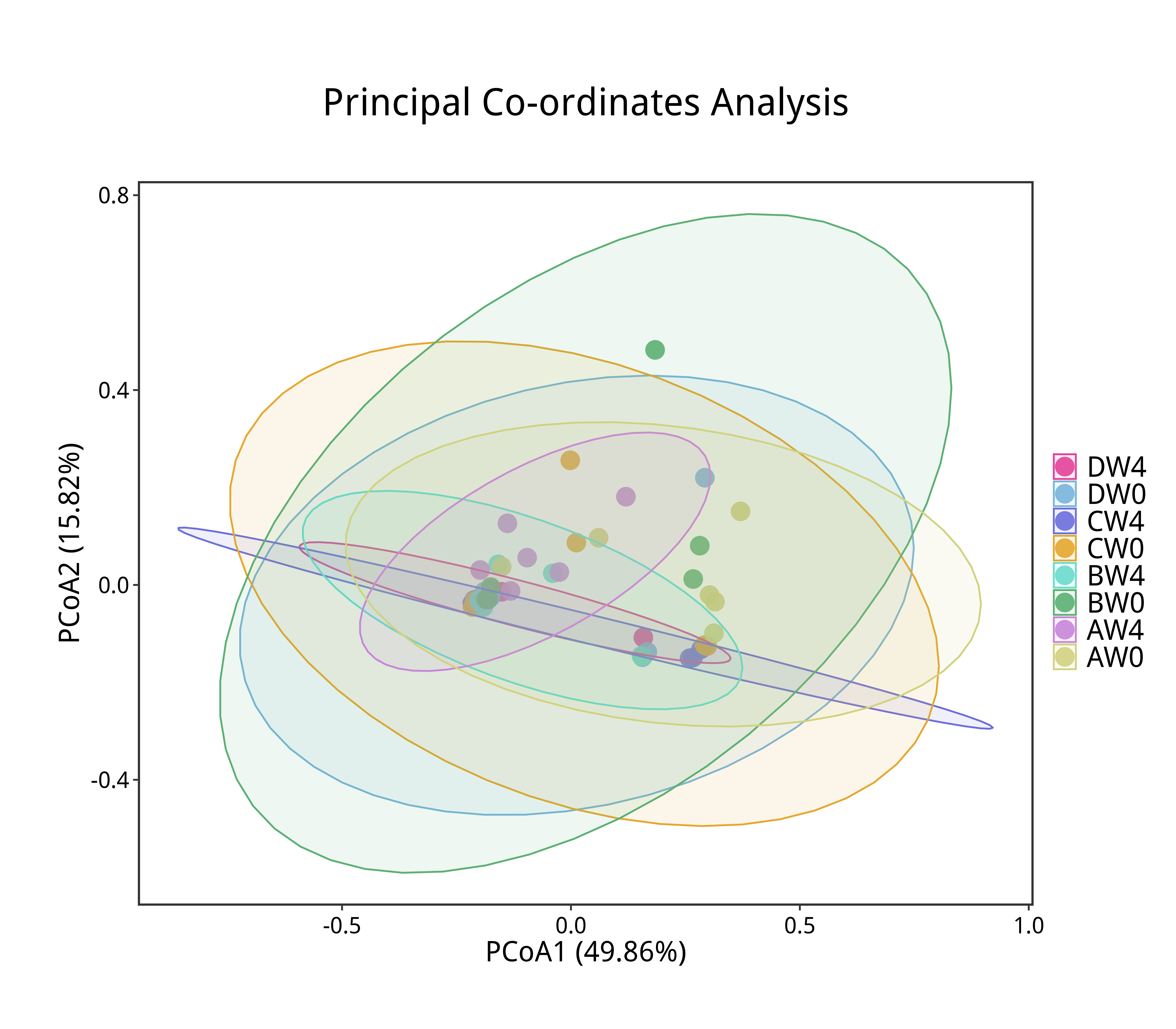

Supplement: Supplementary file 1 [file DataSheet1.zip › summary/summary/5_Beta_diversity/3_PCoA_result/PCoA_2D/Beta_Diversity.weighted_unifrac.PCoA.no_label.png]

# Principal Co-ordinates Analysis

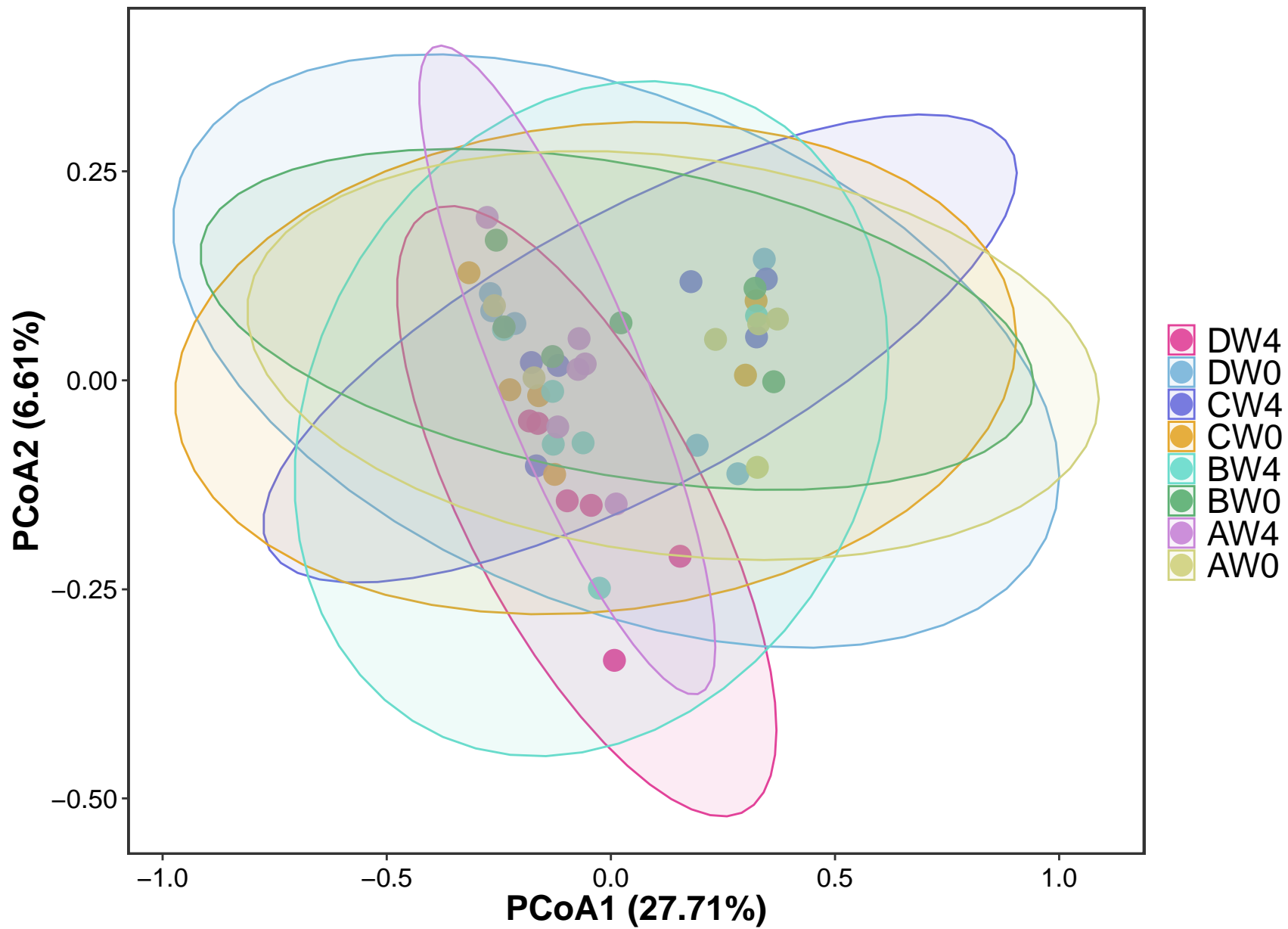

Supplement: Supplementary file 1 [file DataSheet1.zip › summary/summary/5_Beta_diversity/3_PCoA_result/PCoA_2D/Beta_Diversity.unweighted_unifrac.PCoA.no_label.pdf]

# Principal Co-ordinates Analysis

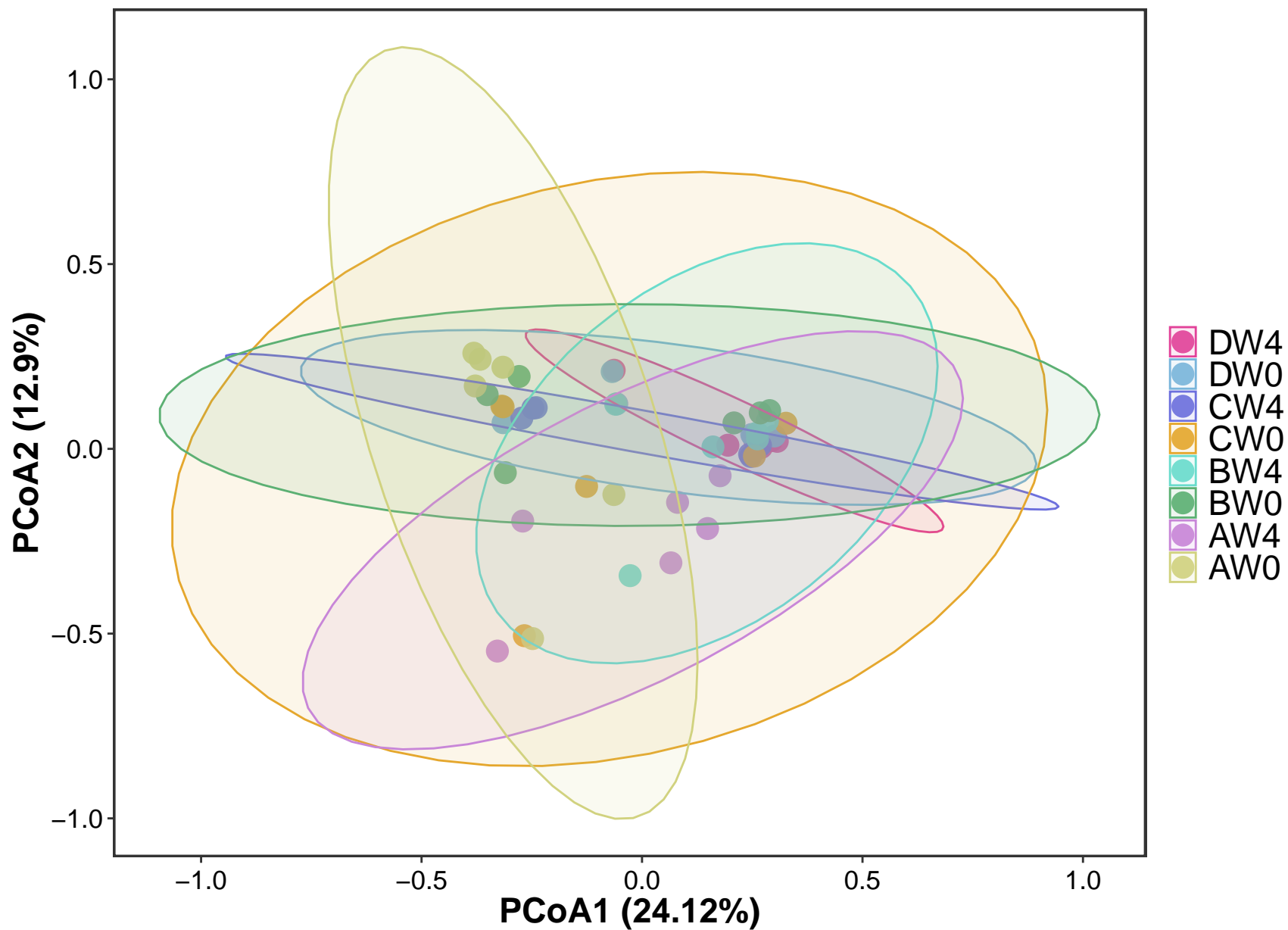

Supplement: Supplementary file 1 [file DataSheet1.zip › summary/summary/5_Beta_diversity/3_PCoA_result/PCoA_2D/Beta_Diversity.bray_curtis.PCoA.no_label.pdf]

# Principal Co-ordinates Analysis

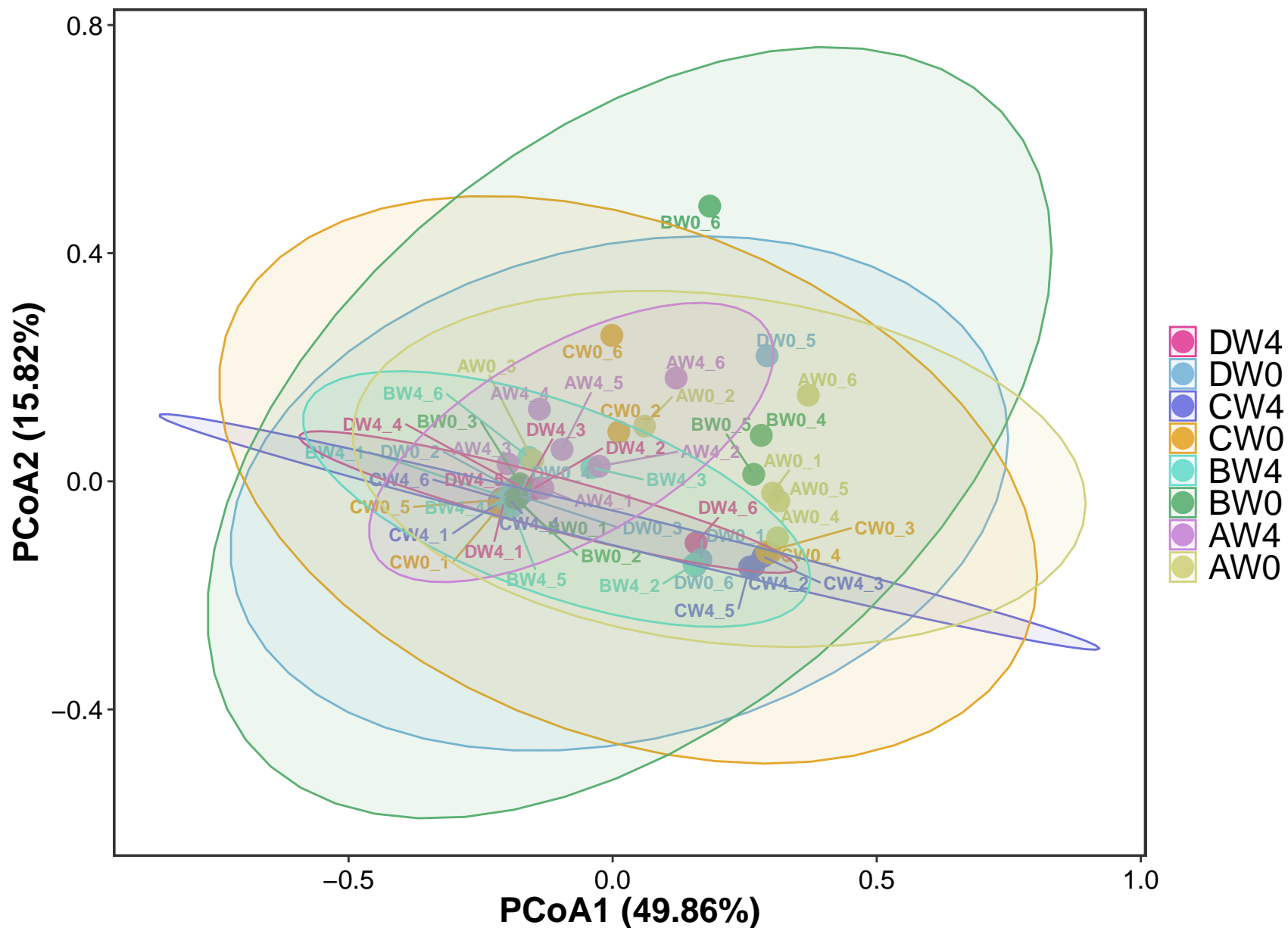

Supplement: Supplementary file 1 [file DataSheet1.zip › summary/summary/5_Beta_diversity/3_PCoA_result/PCoA_2D/Beta_Diversity.weighted_unifrac.PCoA.with_label.pdf]

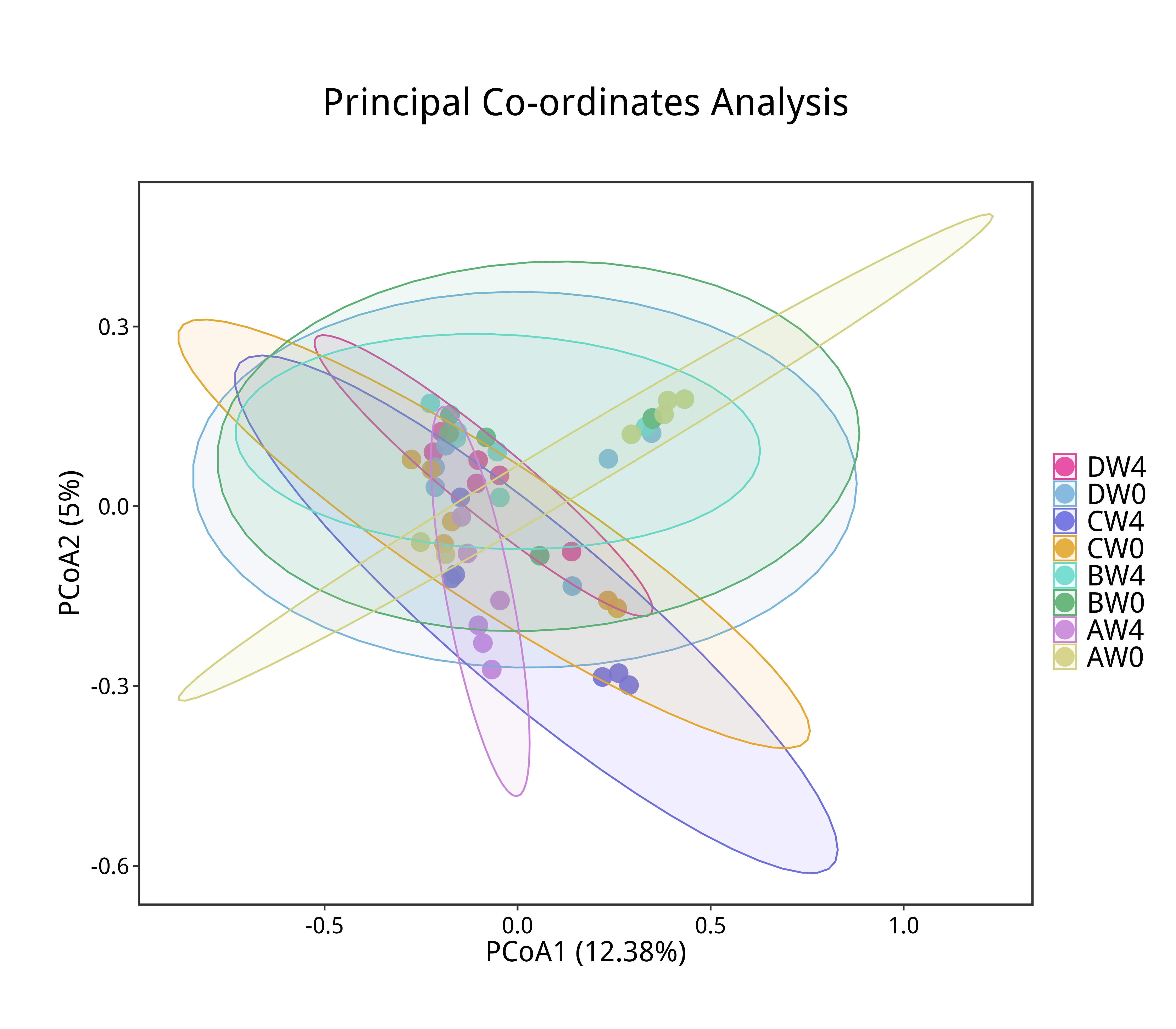

Supplement: Supplementary file 1 [file DataSheet1.zip › summary/summary/5_Beta_diversity/3_PCoA_result/PCoA_2D/Beta_Diversity.jaccard.PCoA.no_label.png]

# Principal Co-ordinates Analysis

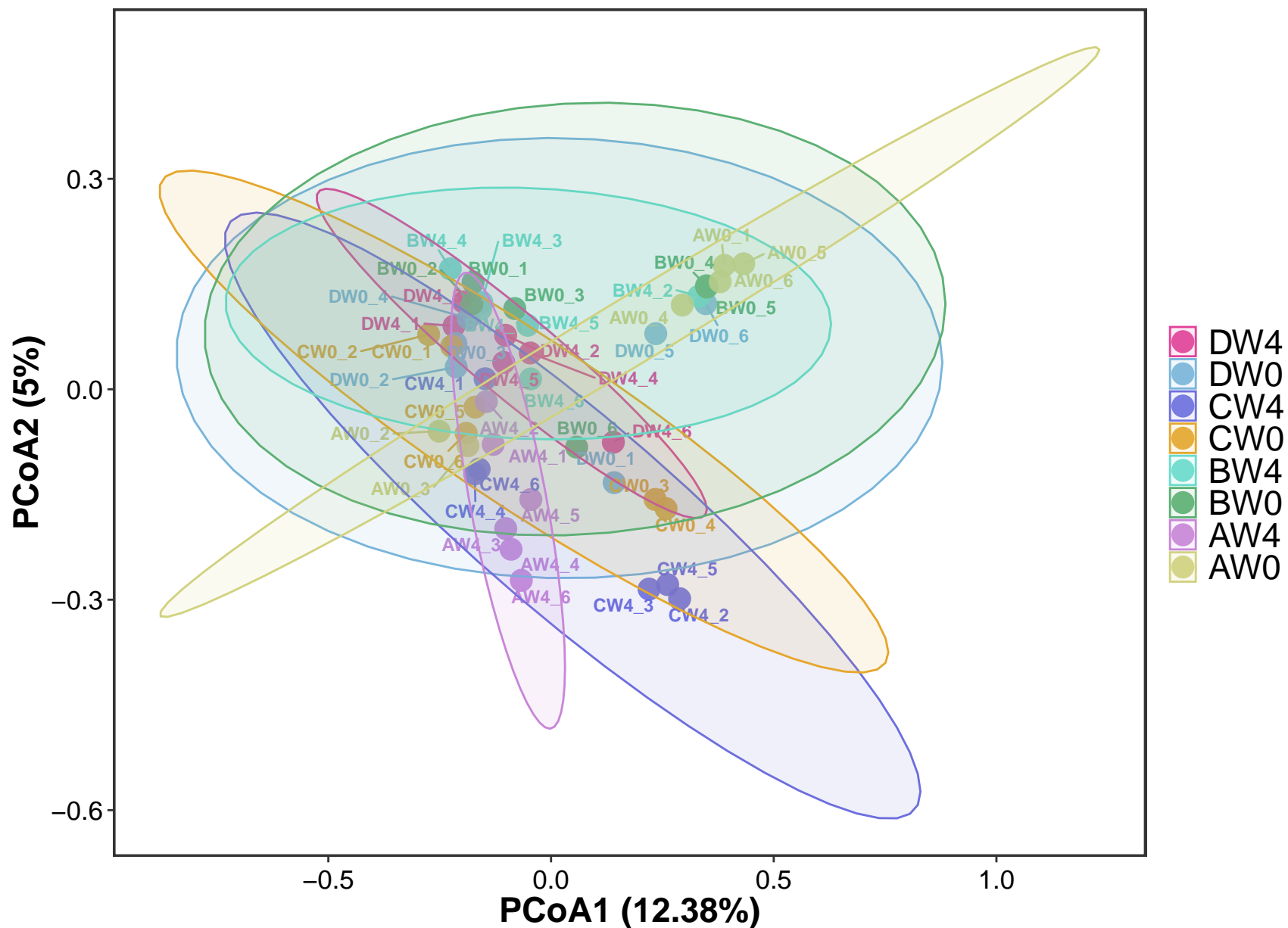

Supplement: Supplementary file 1 [file DataSheet1.zip › summary/summary/5_Beta_diversity/3_PCoA_result/PCoA_2D/Beta_Diversity.jaccard.PCoA.with_label.pdf]

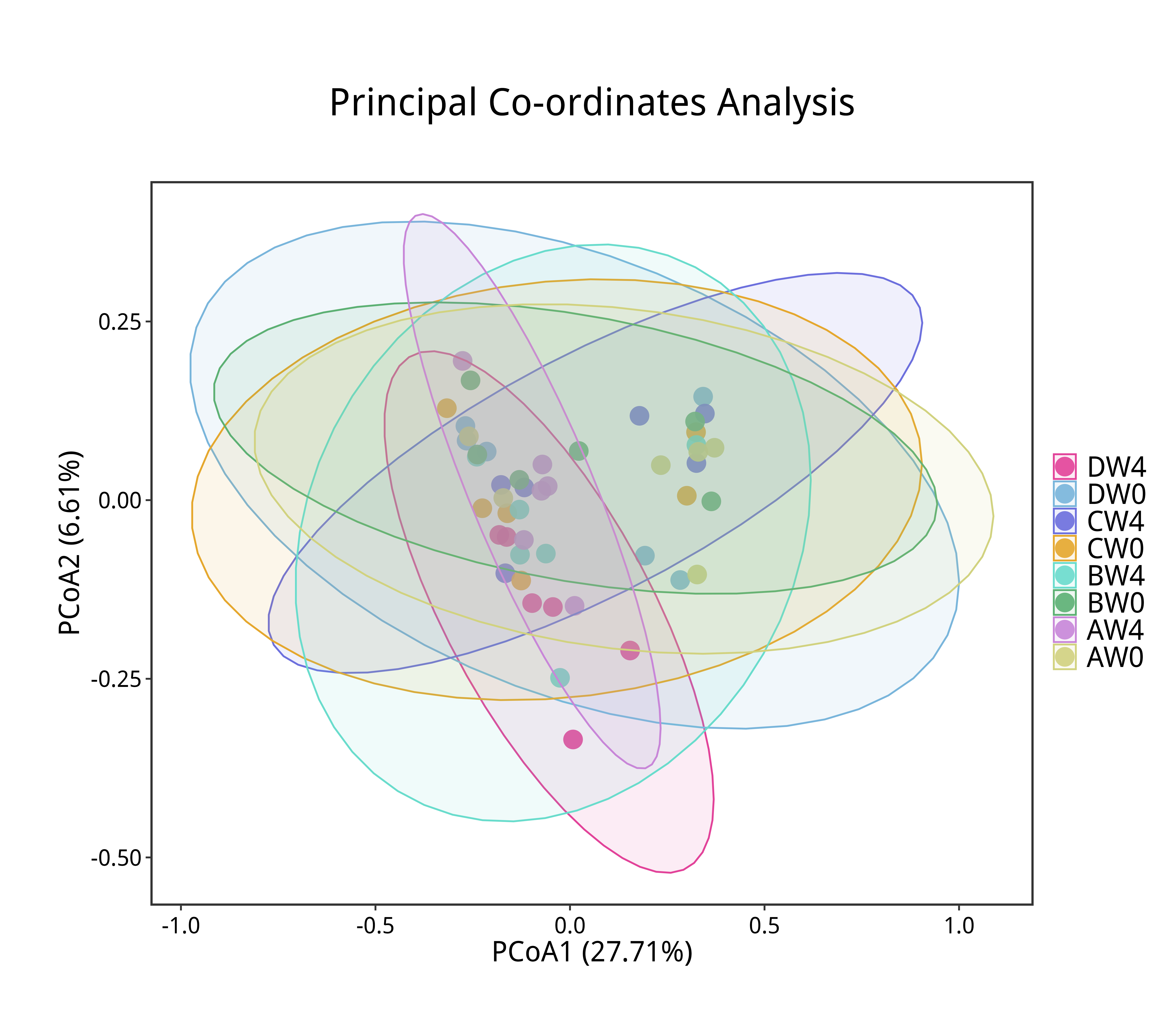

Supplement: Supplementary file 1 [file DataSheet1.zip › summary/summary/5_Beta_diversity/3_PCoA_result/PCoA_2D/Beta_Diversity.unweighted_unifrac.PCoA.no_label.png]

# Principal Co-ordinates Analysis

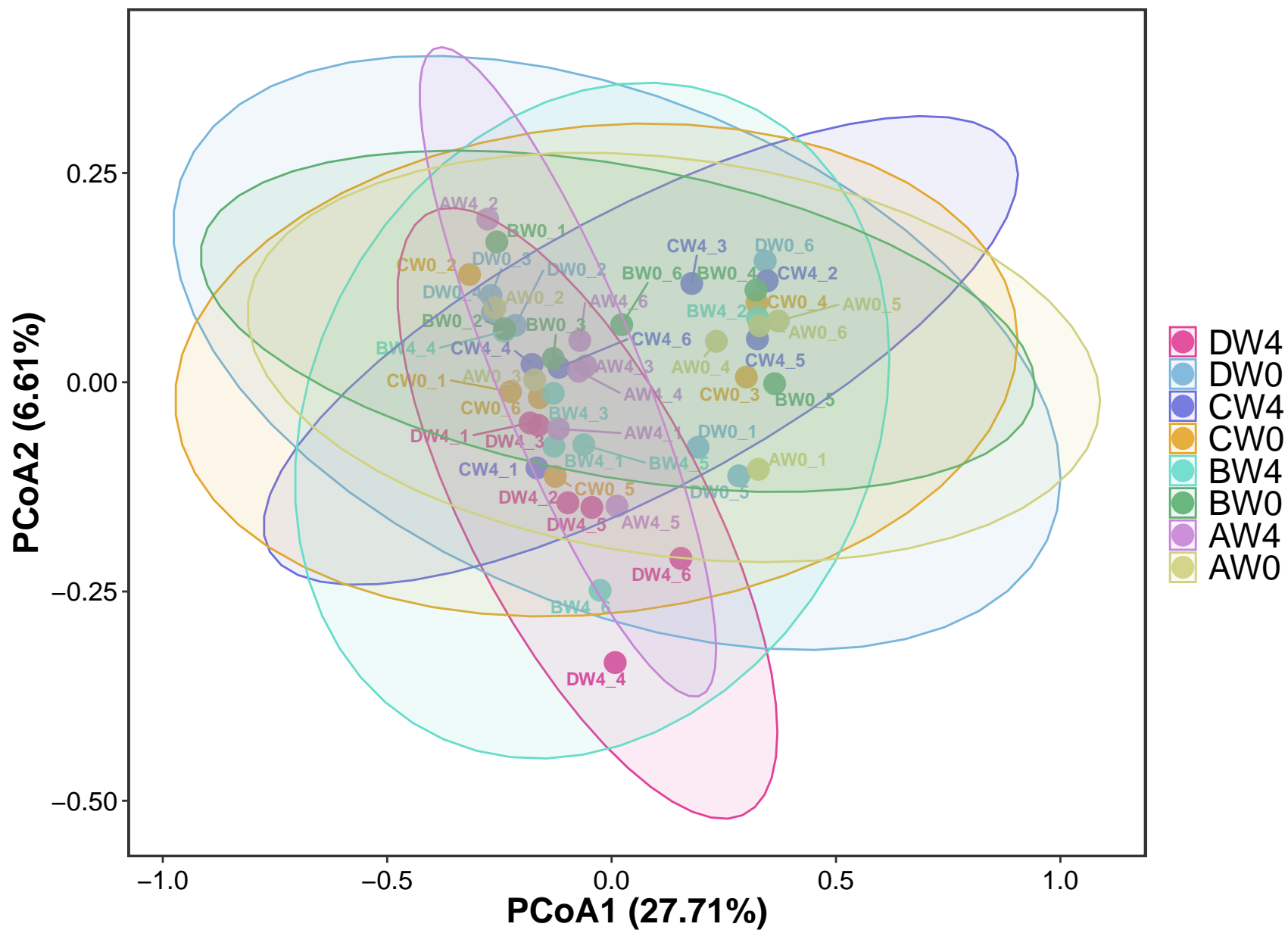

Supplement: Supplementary file 1 [file DataSheet1.zip › summary/summary/5_Beta_diversity/3_PCoA_result/PCoA_2D/Beta_Diversity.unweighted_unifrac.PCoA.with_label.pdf]

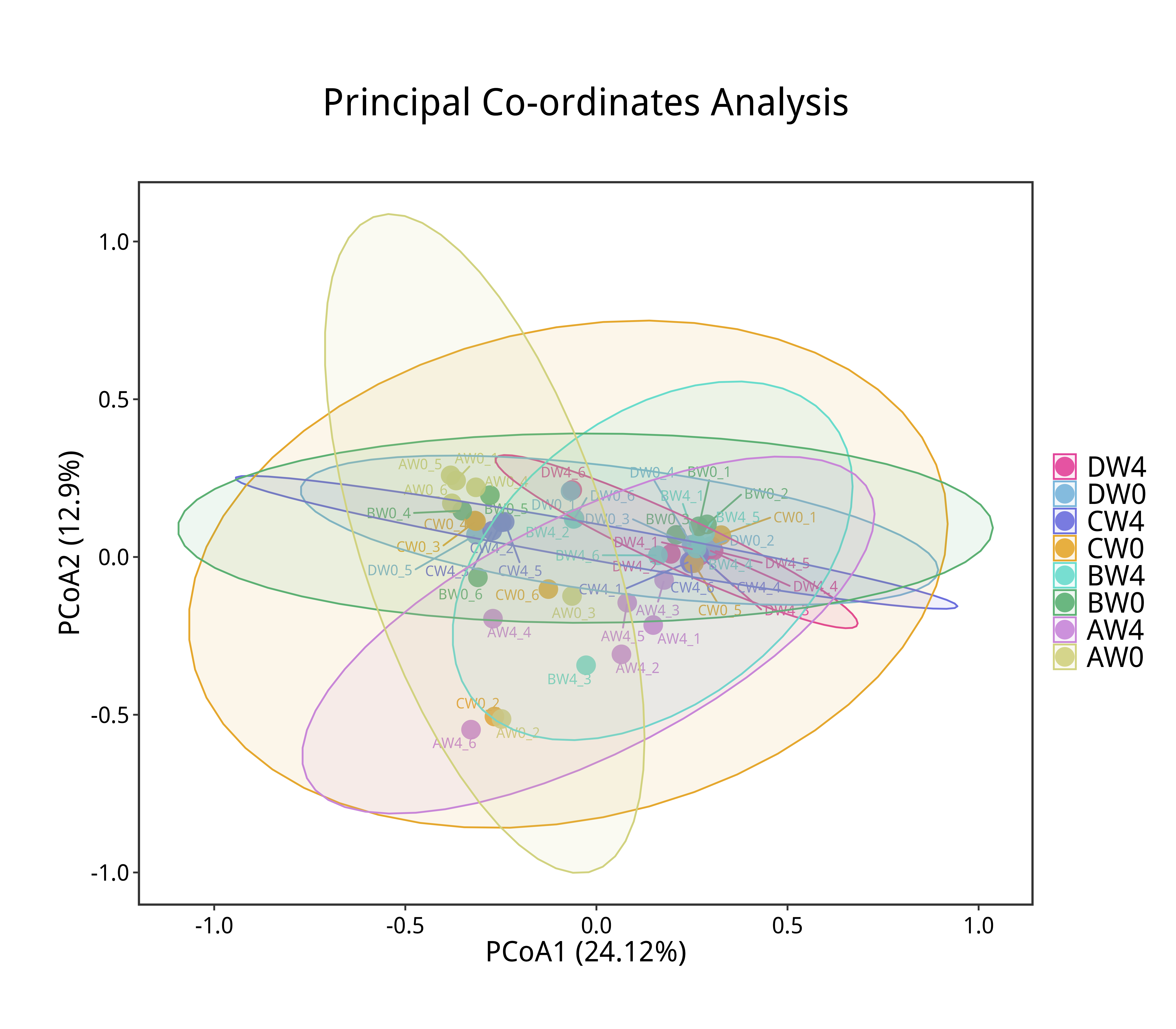

Supplement: Supplementary file 1 [file DataSheet1.zip › summary/summary/5_Beta_diversity/3_PCoA_result/PCoA_2D/Beta_Diversity.bray_curtis.PCoA.with_label.png]

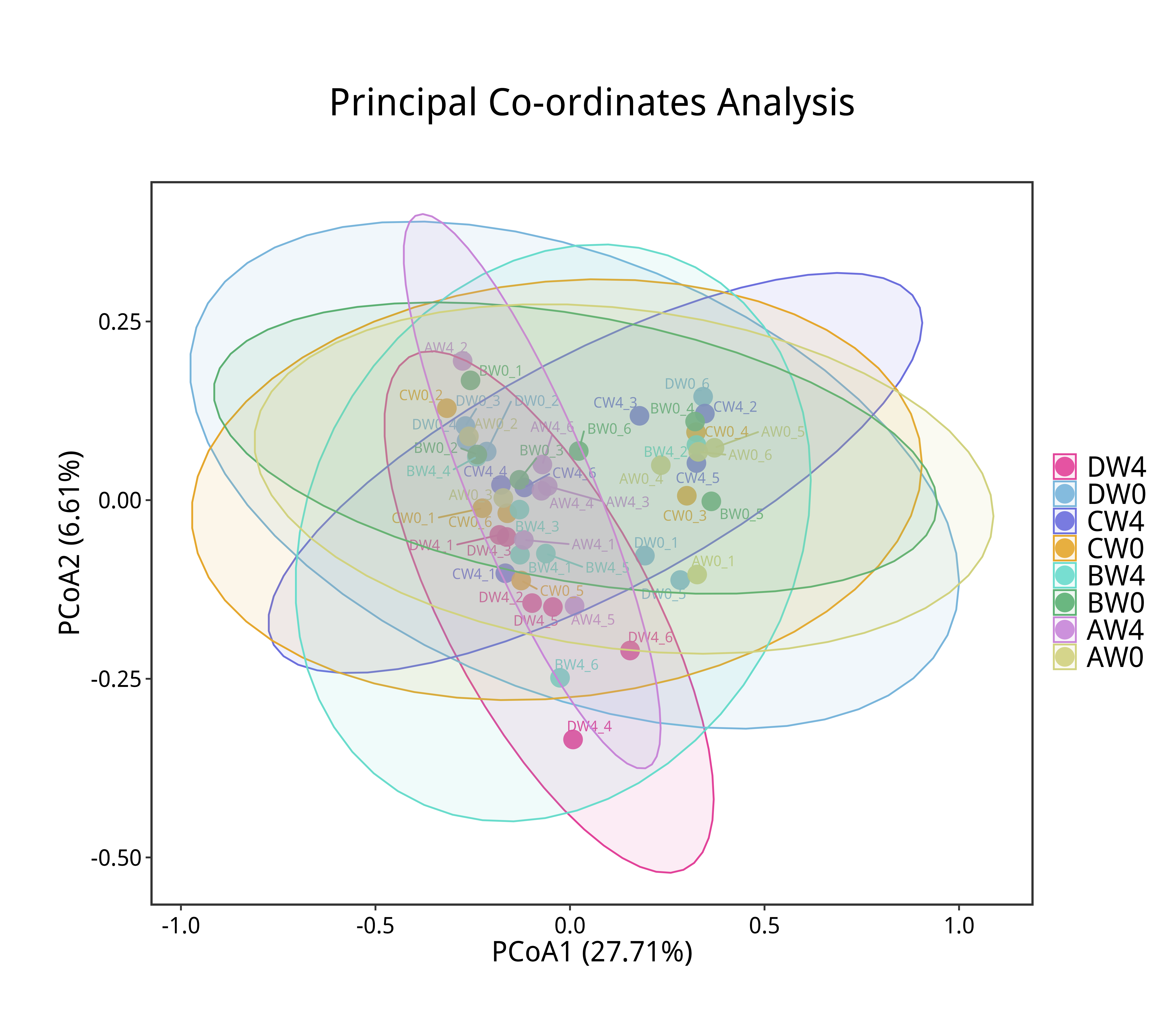

Supplement: Supplementary file 1 [file DataSheet1.zip › summary/summary/5_Beta_diversity/3_PCoA_result/PCoA_2D/Beta_Diversity.unweighted_unifrac.PCoA.with_label.png]

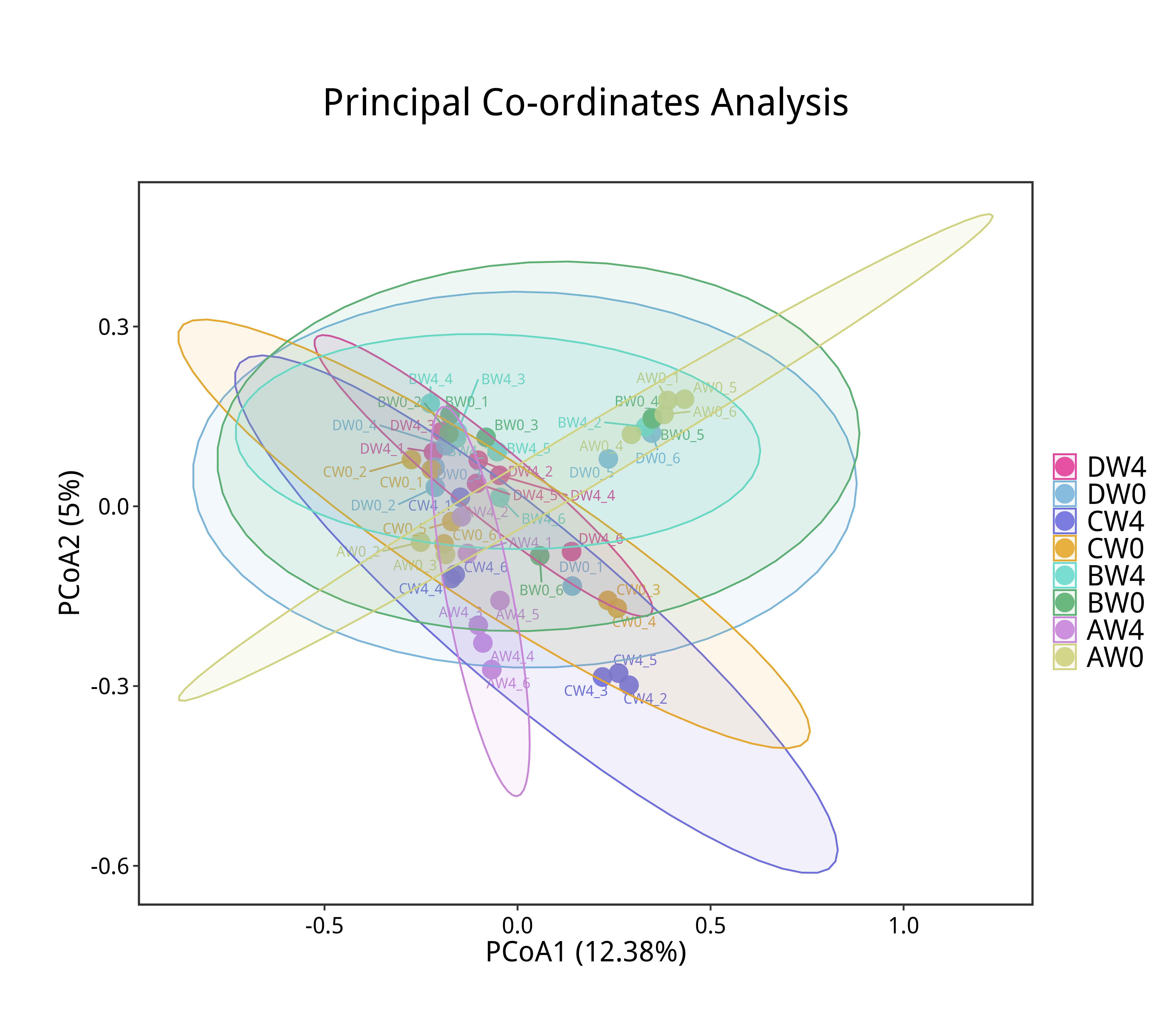

Supplement: Supplementary file 1 [file DataSheet1.zip › summary/summary/5_Beta_diversity/3_PCoA_result/PCoA_2D/Beta_Diversity.jaccard.PCoA.with_label.png]

# Principal Co-ordinates Analysis

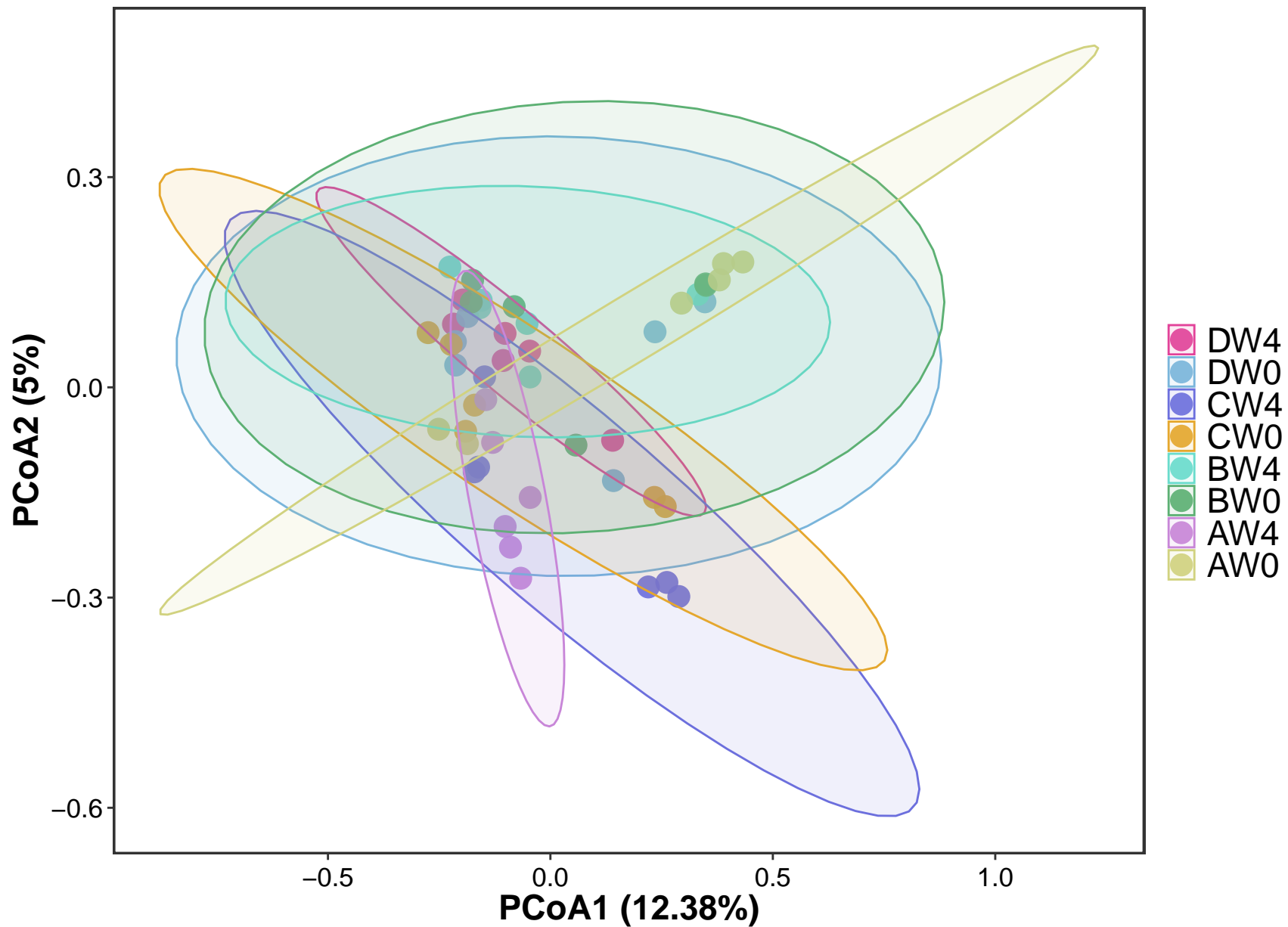

Supplement: Supplementary file 1 [file DataSheet1.zip › summary/summary/5_Beta_diversity/3_PCoA_result/PCoA_2D/Beta_Diversity.jaccard.PCoA.no_label.pdf]

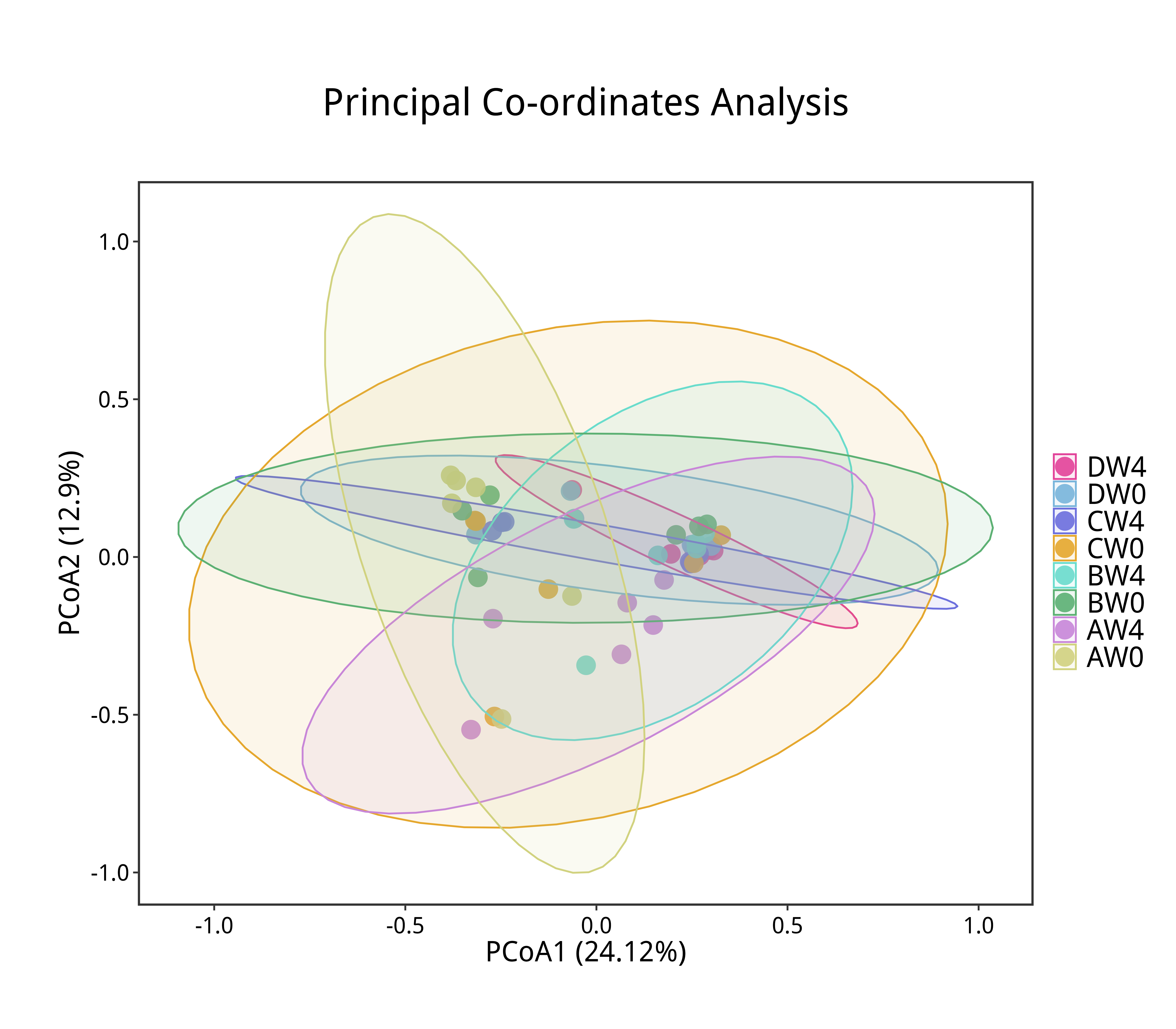

Supplement: Supplementary file 1 [file DataSheet1.zip › summary/summary/5_Beta_diversity/3_PCoA_result/PCoA_2D/Beta_Diversity.bray_curtis.PCoA.no_label.png]

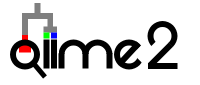

Supplement: Supplementary file 1 [file DataSheet1.zip › summary/summary/5_Beta_diversity/3_PCoA_result/PCoA_3D/weighted_unifrac_pcoa_html/q2templateassets/img/qiime2-rect-200.png]

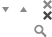

Supplement: Supplementary file 1 [file DataSheet1.zip › summary/summary/5_Beta_diversity/3_PCoA_result/PCoA_3D/weighted_unifrac_pcoa_html/vendor/css/chosen-sprite.png]

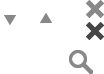

Supplement: Supplementary file 1 [file DataSheet1.zip › summary/summary/5_Beta_diversity/3_PCoA_result/PCoA_3D/weighted_unifrac_pcoa_html/vendor/css/chosen-sprite@2x.png]

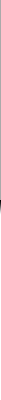

Supplement: Supplementary file 1 [file DataSheet1.zip › summary/summary/5_Beta_diversity/3_PCoA_result/PCoA_3D/weighted_unifrac_pcoa_html/vendor/css/images/ui-bg_glass_75_dadada_1x400.png]

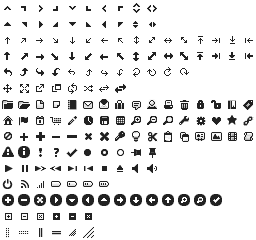

Supplement: Supplementary file 1 [file DataSheet1.zip › summary/summary/5_Beta_diversity/3_PCoA_result/PCoA_3D/weighted_unifrac_pcoa_html/vendor/css/images/ui-icons_222222_256x240.png]

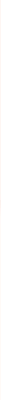

Supplement: Supplementary file 1 [file DataSheet1.zip › summary/summary/5_Beta_diversity/3_PCoA_result/PCoA_3D/weighted_unifrac_pcoa_html/vendor/css/images/ui-bg_glass_95_fef1ec_1x400.png]

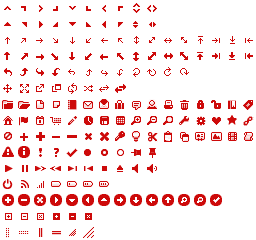

Supplement: Supplementary file 1 [file DataSheet1.zip › summary/summary/5_Beta_diversity/3_PCoA_result/PCoA_3D/weighted_unifrac_pcoa_html/vendor/css/images/ui-icons_cd0a0a_256x240.png]

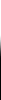

Supplement: Supplementary file 1 [file DataSheet1.zip › summary/summary/5_Beta_diversity/3_PCoA_result/PCoA_3D/weighted_unifrac_pcoa_html/vendor/css/images/ui-bg_highlight-soft_75_cccccc_1x100.png]

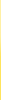

Supplement: Supplementary file 1 [file DataSheet1.zip › summary/summary/5_Beta_diversity/3_PCoA_result/PCoA_3D/weighted_unifrac_pcoa_html/vendor/css/images/ui-bg_highlight-soft_75_ffe45c_1x100.png]

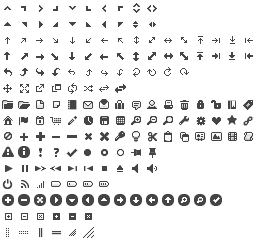

Supplement: Supplementary file 1 [file DataSheet1.zip › summary/summary/5_Beta_diversity/3_PCoA_result/PCoA_3D/weighted_unifrac_pcoa_html/vendor/css/images/ui-icons_454545_256x240.png]

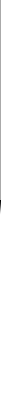

Supplement: Supplementary file 1 [file DataSheet1.zip › summary/summary/5_Beta_diversity/3_PCoA_result/PCoA_3D/weighted_unifrac_pcoa_html/vendor/css/images/ui-bg_glass_75_e6e6e6_1x400.png]

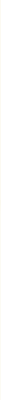

Supplement: Supplementary file 1 [file DataSheet1.zip › summary/summary/5_Beta_diversity/3_PCoA_result/PCoA_3D/weighted_unifrac_pcoa_html/vendor/css/images/ui-bg_glass_55_fbf9ee_1x400.png]

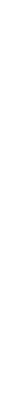

Supplement: Supplementary file 1 [file DataSheet1.zip › summary/summary/5_Beta_diversity/3_PCoA_result/PCoA_3D/weighted_unifrac_pcoa_html/vendor/css/images/ui-bg_glass_65_ffffff_1x400.png]

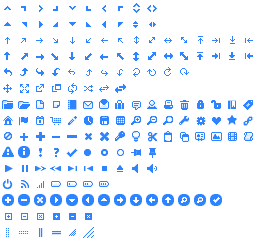

Supplement: Supplementary file 1 [file DataSheet1.zip › summary/summary/5_Beta_diversity/3_PCoA_result/PCoA_3D/weighted_unifrac_pcoa_html/vendor/css/images/ui-icons_2e83ff_256x240.png]

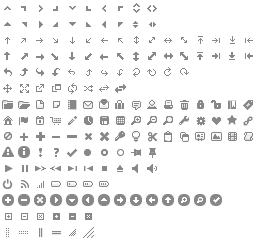

Supplement: Supplementary file 1 [file DataSheet1.zip › summary/summary/5_Beta_diversity/3_PCoA_result/PCoA_3D/weighted_unifrac_pcoa_html/vendor/css/images/ui-icons_888888_256x240.png]

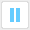

Supplement: Supplementary file 1 [file DataSheet1.zip › summary/summary/5_Beta_diversity/3_PCoA_result/PCoA_3D/weighted_unifrac_pcoa_html/img/pause.png]

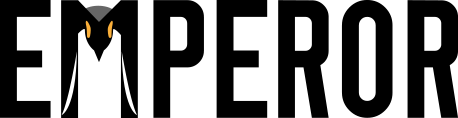

Supplement: Supplementary file 1 [file DataSheet1.zip › summary/summary/5_Beta_diversity/3_PCoA_result/PCoA_3D/weighted_unifrac_pcoa_html/img/emperor.png]

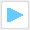

Supplement: Supplementary file 1 [file DataSheet1.zip › summary/summary/5_Beta_diversity/3_PCoA_result/PCoA_3D/weighted_unifrac_pcoa_html/img/play.png]

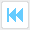

Supplement: Supplementary file 1 [file DataSheet1.zip › summary/summary/5_Beta_diversity/3_PCoA_result/PCoA_3D/weighted_unifrac_pcoa_html/img/reset.png]
